# Lignin Biopolymers in the Age of Controlled Polymerization

**DOI:** 10.3390/polym11071176

**Published:** 2019-07-12

**Authors:** Mitra S. Ganewatta, Hasala N. Lokupitiya, Chuanbing Tang

**Affiliations:** 1 Department of Chemistry and Biochemistry, University of South Carolina, Columbia, SC 29208, USA; 2 Ingevity Corporation, 5255 Virginia Avenue, North Charleston, SC 29406, USA; 3 Department of Chemistry and Biochemistry, College of Charleston, 66 George Street, Charleston, SC 29424, USA

**Keywords:** sustainable polymers, biomass, lignin, controlled polymerization

## Abstract

Polymers made from natural biomass are gaining interest due to the rising environmental concerns and depletion of petrochemical resources. Lignin isolated from lignocellulosic biomass is the second most abundant natural polymer next to cellulose. The paper pulp process produces industrial lignin as a byproduct that is mostly used for energy and has less significant utility in materials applications. High abundance, rich chemical functionalities, CO_2_ neutrality, reinforcing properties, antioxidant and UV blocking abilities, as well as environmental friendliness, make lignin an interesting substrate for materials and chemical development. However, poor processability, low reactivity, and intrinsic structural heterogeneity limit lignins′ polymeric applications in high-performance advanced materials. With the advent of controlled polymerization methods such as ATRP, RAFT, and ADMET, there has been a great interest in academia and industry to make value-added polymeric materials from lignin. This review focuses on recent investigations that utilize controlled polymerization methods to generate novel lignin-based polymeric materials. Polymers developed from lignin-based monomers, various polymer grafting technologies, copolymer properties, and their applications are discussed.

## 1. Introduction

Lignin is a heterogenous phenylpropanoid macromolecule with a three-dimensionally branched architecture composed of random crosslinks of monomeric units called monolignols. This random and complex architecture of lignin, comprising of a matrix of phenolic and aliphatic substances awards it to become one of the most recalcitrant biopolymers, an ideal characteristic for structural support for many species and a defensive barrier against co-evolving pathogens and herbivores [1]. In addition, lignin acts as a permanent binding agent between cells, energy storage, an antioxidant, a UV blocker, and a hydrophobic agent in plants. Lignification is a major sink for carbon in plants and fixes a large amount of atmospheric carbon. Upon the death of plants, lignin undergoes natural biodegradation by soil microorganisms resulting in the formation of soil organic matter. Therefore, lignin formation has a significant contribution to the physiology of vascular plants, the carbon cycle and the ecological balance of the Earth.

The limitations of non-renewable petroleum-based chemicals and grave concerns regarding environmental pollution are urging many citizens and nations for the utilization of renewable and environment-friendly feedstocks to replace current petroleum-based materials [2]. Lignin has gained much attention as a valuable raw natural resource for energy, chemicals and materials space. Lignin is the second most abundant organic substance in the world and has been estimated to represent 30% of the total biomass produced in the biosphere. It is estimated that the total lignin present in the biosphere exceeds 300 billion tons and 20 billion tons of lignin is produced annually through biosynthesis [3,4]. In addition, lignin boasts the qualities of a non-food biomass feedstock and an environmentally friendly biodegradable material. In plant cells, biological molecules including cellulose, hemicellulose, and lignin are bound tightly together forming rigid cell wall structures and comprise the lignocellulose biomass that must be broken down to isolate lignin. Millions of tons of lignin are produced by the paper pulping industry every year and are mostly (>95%) treated as waste or used in low-value applications such as fuels. Commercially used lignin is typically produced as lignosulfonates or kraft lignins. The advances of biorefineries that convert cellulosic biomass into liquid transportation fuels will eventually create a surplus of industrial lignin [5]. Chemical corporations such as Ingevity Corporation has been producing high-quality kraft lignin, i.e., Indulin AT, for many decades. In addition, the Domtar corporation in North Carolina and Stora Enso′s Sunila mill in Finland recently started producing LignoBoost™ kraft lignin. Borregaard and Rayonier started a new venture, Lignotech Florida, which produces lignosulfonates. With the increased capacity to produce lignins, the prospects of specialty chemicals originating from industrial lignin is substantially increasing. Lignin-based materials and chemicals represent potential value-added products as (1) macromolecule additives or polymer blends; (2) fragmented aromatic compounds such as benzene, toluene, xylenes; (3) carbon materials [4]. About 2% of all lignin generated in paper production is isolated, modified and sold in the chemicals market. Currently, lignin is utilized in applications including dispersant for agricultural chemicals, oil well drilling, dyestuffs, carbon black, cement, gypsum, etc., emulsifiers, lead storage batteries, phenolic resins, binders and pelleting aids, bricks, ceramics, dust control, asphalt, water treatment, heavy metal sequestrant, vanillin production, etc. The global lignin market was valued at approximately USD 775 million in 2014 and is expected to go beyond USD 906 million in 2025 [6,7]. New commercialization opportunities of up to $242 billion are emerging [8,9].

The absence of a unique and a well-defined structure with certain characteristic properties and functionalities make it challenging to produce value-added lignin products with consistent qualities. Poor processability, low reactivity, brittleness, and intrinsic structural heterogeneity limit lignins′ polymeric applications in high-performance advanced materials. Therefore, aimed at value-added advanced applications, the structure and properties of lignin, and avenues to fine-tune its properties need to be further investigated. Lignin-based polymeric materials can be developed by blending lignin with commercial off the shelf polymers. Benefits such as improved thermal and mechanical properties for such blends have been reported. However, the immiscibility of lignin with most polymers limits such materials development [10]. Lignin graft polymers are one avenue to improve the compatibility of lignin with matrix polymers. In addition, lignin can be utilized as a stiff, macromolecular backbone to prepare sustainable thermoplastic elastomers and mechanically robust materials [11]. The interest in developing lignin graft polymers has generated many recent publications. Lignin-derived monomers can also be used to construct well-defined polymers with tailor-made properties. This review attempts to gather recent developments in lignin polymer chemistry with a focus on controlled polymerization methods.

## 2. Lignin Structure and Composition

**Figure 1:**
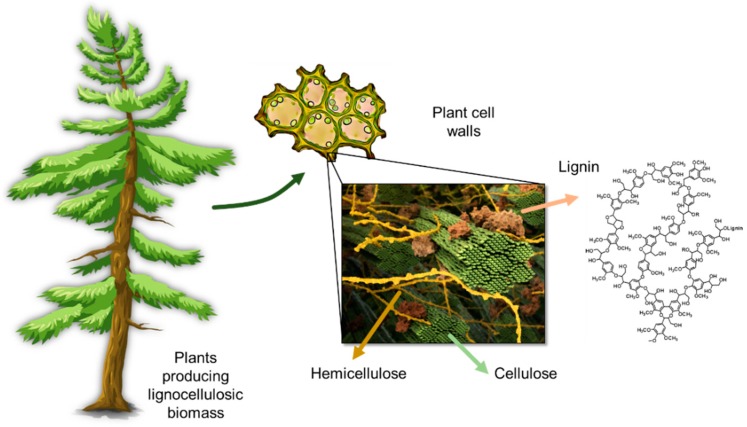
Schematic representation of the lignin location and structure in plants. Part of the image was kindly provided by scistyle.com and Jeremy C. Smith et al. UT/Oak Ridge National Laboratory Center for Molecular Biophysics.

**Figure 2:**
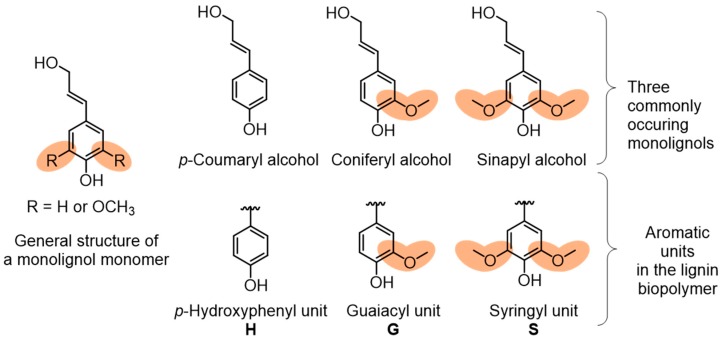
Monolignol building blocks and the resulting aromatic units present in lignin.

Lignocellulosic biomass contains cellulose fibers that are locked into the cell wall structures in a matrix composed of lignin and hemicellulose (Figure 1). Lignin content and structure in plants vary, depending on the species type, geographic location, tissue of the plant, and numerous other factors. The complex three-dimensional structure of lignin is an outcome of the polymerization of three phenylpropane units that originate from three aromatic alcohols: *p*-coumaryl alcohol, coniferyl alcohol, and sinapyl alcohol (Figure 2).

Typically, softwoods have higher lignin content than hardwoods, where the former has a fraction of 25–35% and the latter 15–25%. In contrast, grasses contain about 10% of lignin and even lower content of 3% in annual plants. Monolignol structural units of hydroxycinnamyl alcohols, i.e., *p*-coumaryl alcohol, coniferyl alcohol, and sinapyl alcohol, undergo enzymatic radical coupling polymerization to produce lignin. It has been identified that peroxidase-mediated dehydrogenation of monolignol units results in a heterogeneous structure of lignin by the formation of C–C bonds and aryl ether linkages. These hydroxycinnamyl alcohols are commonly referred to as *p*-hydroxyphenyl (H), guaiacyl (G) and syringyl (S) units within the lignin structure (Figure 2). Plants contain varying amount of these monolignol units. Softwood lignins have an abundance of G units, and hardwood has both G and S units with a more complex structure. Non-woody species such as grasses contain a substantial amount of H units.

**Figure 3:**
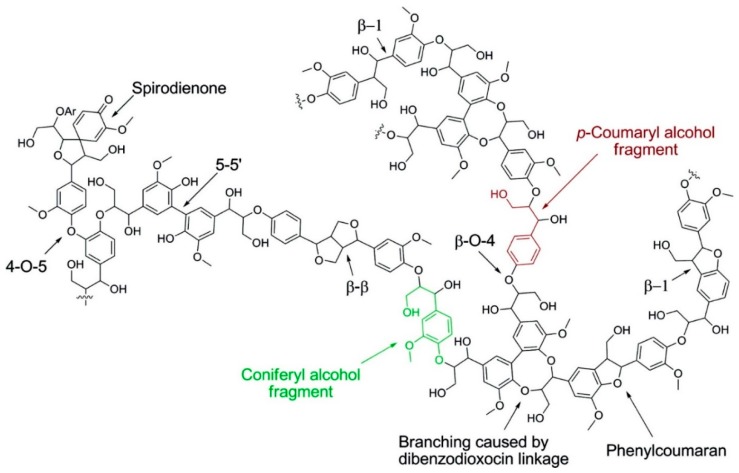
A schematic representation of softwood lignin structure. Reproduced with permission [12].

A schematic representation of the softwood lignin structure showing common linkages is illustrated in Figure 3. It can be noted that lignin macromolecules contain complex covalent linkages with various types of C–O bonds and C–C bonds. The chemical and physical properties greatly depend on lignin inter- and intramolecular interactions and its solution conformation. Lignin has a glassy and hard structure at room temperature and softens above its glass transition temperature (*T*_g_). Further heating (>250 °C) results in decomposition producing charcoal, tar and small molecular fragments.

**Figure 4:**
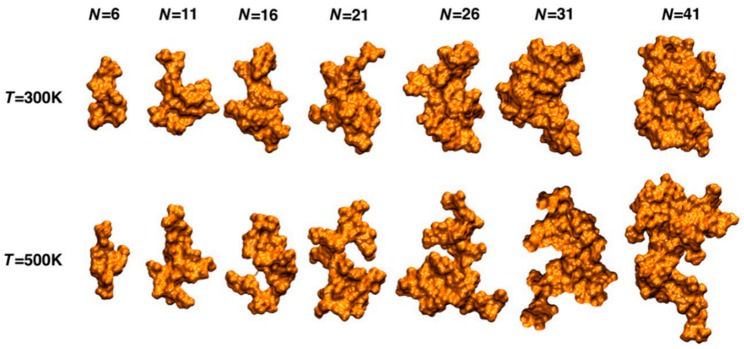
Representative structures of the atomistic molecular dynamics simulations of lignin in aqueous solution at degrees of polymerization between 6 and 41 at two temperatures. Reproduced with permission [13].

According to molecular dynamics simulation investigations, Smith et al. report that lignin in water adopts a collapsed conformation (Figure 4) [13]. The structure transition from a mobile, extended to a glassy, compact state with decreasing temperatures [14].

## 3. Extraction of Lignin

Industrially, lignin is dismantled from cellulosic fibers by chemical treatment processes, which break down lignin-carbohydrate complexes. During this process, partial depolymerization of the complex lignin macromolecules occurs, resulting in smaller fractions that facilitate its solubilization. Due to the presence of various reactive sites, re-polymerization (condensation) also occurs within the matrix, forming stronger C–C bonds, and leading to alteration of the native lignin structure. Owing to feedstock variability and extraction methods cause the isolated lignin to have diverse structures and a range of physical properties. Paper pulping industry and biorefineries currently contribute to the commercial production of technical lignins. About 50 million metric tons of lignin is produced by the pulp and paper industry and most of it is combusted as a fuel for the generation of energy used to operate the paper mills. Only about 2% of that volume comes into the chemicals market. There are several excellent reviews that summarize lignin extraction methods [12,15,16,17,18,19]. Therefore, this review only briefly mentions some prominent methods and recent developments. Table 1 summarizes major lignin extractions methods.

### 3.1. Ionic Liquids for Lignin Extraction 

Ionic liquids (ILs) are salts that are in the liquid state at ambient temperatures (<100 °C). ILs have gained much attention in recent literature as good solvents for cellulose and lignin processing [25]. ILs have superior dissolution capabilities, thermal stability, low volatility, and low flammability which makes them great candidates for lignin extraction. However, the expensive nature of ILs makes them challenging to use in industry, and complete removal of ILs from recovered lignin remains a challenge. 1-Ethyl-3-methylimidazolium acetate ((Emim)(OAc)) and 1-butyl-3-methylimidazolium chloride ([Bmim]Cl) are two of the frequently used ILs for biomass dissolution [26] Owing to the strong hydrogen-bonding basicity of certain ions, ILs can easily dissolve biomass including lignin structures. Dissolution followed by the usage of selective anti-solvents such as water, acetone, acetonitrile, etc., or combinations thereof can precipitate and isolate biomass components.

### 3.2. Lignin-First Method and Biorefinery Concepts

During the paper pulping process, where lignin is considered a waste material, lignin is prone to irreversible degradation and rearrangement, resulting in recalcitrant condensed aromatic structures with poor functionality for materials development. To circumvent this issue, and treating lignin valorization as a priority, a novel biorefinery approach called the “lignin-first” method has gained much interest in the scientific community [27]. In this approach, two strategies are actively involved to prevent unnecessary lignin degradation and condensation during the fractionation process: (i) tandem depolymerization–stabilization of native lignin, and (ii) active preservation of β-O-4 bonds. Lignocellulosic biorefineries use pretreatments such as dilute acids or hydrothermal methods to enable enzymes at later stages to efficiently break down cellulose and other polysaccharides. Enzymatic hydrolysis affords lignin-rich streams.

## 4. Chemical Modifications of Lignin

**Figure 5:**
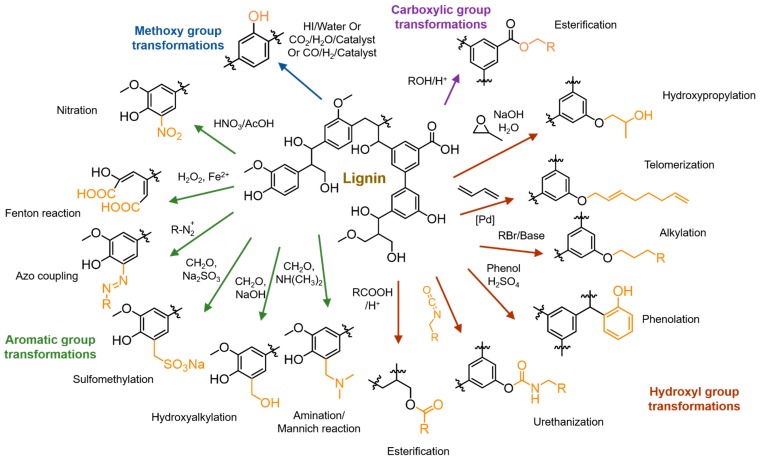
A summary of chemical trasnformations to diversify chemical functionality on lignin.

Lignin downstream modifications and applications obviously require proper chemical handles. Natural lignin has aromatic sites, alcohols, methoxyl, carboxyl, and carbonyl functionalities. However, the limited reactivities and subpar physical properties encourage chemists to install novel functional groups on lignin. In this section, current advances in chemical modifications of lignin macromolecules to introduce useful functionalities via small molecule-based transformations are discussed. Several reviews have concisely discussed such transformations [20,28]. Figure 5 summarizes several strategies that introduce new chemical reactive sites or functionalities on lignin macromolecules. Traditionally, lignin hydroxyl groups and aromatic units are heavily used for chemical functionalization. The presence of aromatic and aliphatic hydroxyl groups facilitates chemical reactions such as esterification, alkylation or etherification, hydroxypropylation with epoxides, urethanization via isocyanate or non-isocyanate routes. Recently, Sauthier et al. reported kraft lignin functionalization with octadienyl ether linkages through the palladium-catalyzed telomerization of 1,3-butadiene [29]. Lignin phenolation is of interest due to the ability to increase reactive sites for aromatic substitution reactions. Chemical reactions on the aromatic units include sulfomethylation, hydroalkylation, amination, and nitration. Azo coupling reactions were reportedly used to modify lignin properties [30,31]. Kent et al. reported the efficient conversion of lignin into a water-soluble polymer by a chelator-mediated Fenton reaction [32]. Understanding such chemistry will be beneficial to designing modified lignins with desirable properties, as well as new grafting chemistries for polymeric modifications.

## 5. Lignin-Derived Polymers

### 5.1. Overview 

**Figure 6:**
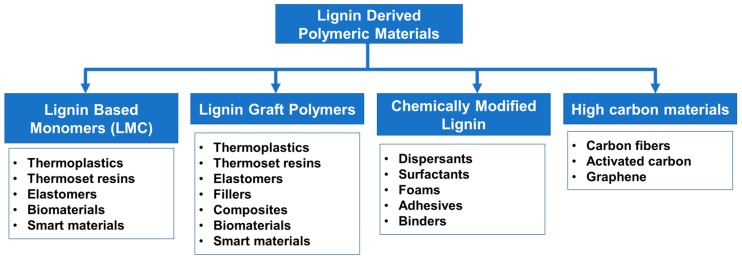
Lignin valorization via polymeric transformations.

Lignin has tremendous potential as a raw material for future material development. Large-scale use of biomass-derived polymers and chemicals are essential for sustainable development and environmental preservation. Current lignin applications in low-end markets such as fuels or cement additives will be saturated with the generation of an excessive amount of lignin streams from biorefineries in addition to paper pulp manufacturing facilities. The unique chemical functionalities and physical properties of lignin make it a potential candidate to develop new polymeric materials. Lignin derivatization using polymer chemistry has become a valuable path to improve its thermo-mechanical properties, as well as chemical functionalization to achieve specialty uses. Lignin valorization via polymeric transformations can be represented into several categories as illustrated in Figure 6. Lignin-derived polymers obtained from monomers to polymer strategy and lignin graft polymers are given focus in this review and commercially used lignin products such as dispersants and high carbon materials are not discussed in detail.

The controlled polymerization methods enable control of polymer compositions, architectures, and functionalities, allowing the development of novel materials with tailored physical and chemical properties. Atom transfer radical polymerization (ATRP) [33], nitroxide mediated polymerization (NMP) [34], reversible addition fragmentation chain transfer (RAFT) [35], ring-opening polymerization (ROP), ring-opening metathesis polymerization (ROMP) [36] and acyclic diene metathesis (ADMET) [37] polymerization are representative examples of such versatile methods to produce well-defined polymers with controlled molecular weight, narrow molecular weight distribution and site-specific functionality. In addition, they allow the generation of high grafting densities on graft polymers and control over end-groups. While there are many reports that investigate the utilization of such methods to make lignin-based biopolymers, it should be noted that there is certainly room for further exploration.

### 5.2. Strategies on Lignin-Based Polymer Synthesis

**Figure 7:**
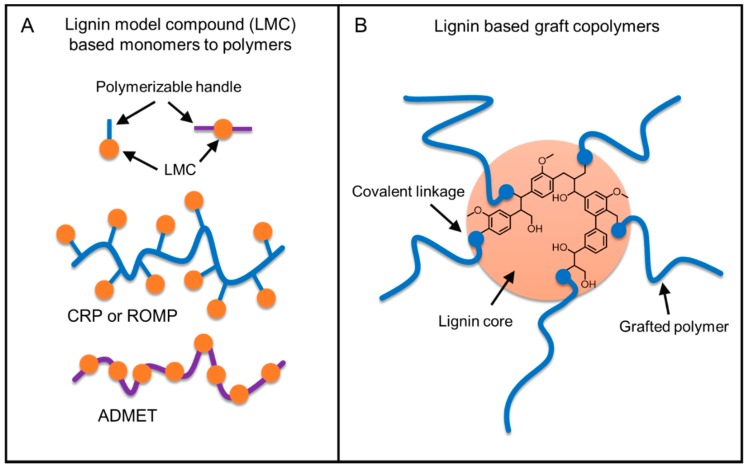
Strategies for lignin-based polymer synthesis.

There are two major routes for the generation of lignin-based polymers (Figure 7). Producing well-defined polymers with greater control of polymer architecture and tunable properties is realized by a bottom-up method where functional monomers are developed from phenolic lignin model compounds (LMC). The other route is to graft polymers using lignin biopolymer as a core unit. Industrially, the latter method is more practical for producing commodity materials with current technologies in a cost-effective way.

### 5.3. From Lignin Model Compounds (LMC) to Novel Biobased Polymers

**Figure 8:**
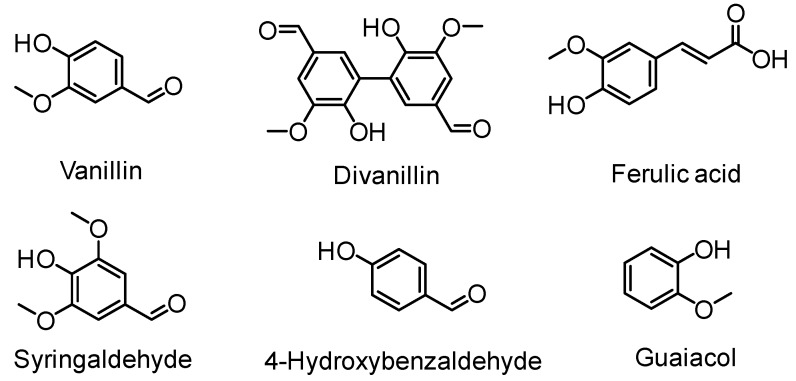
Lignin-derived renewable monomer precursors.

Aromatic compounds fall into a class of essential platform chemicals utilized to manufacture commodity and advanced materials. Rigidity, thermal stability, chemical resistance, hydrophobicity and fire resistance are some of the important properties exerted by aromatic compounds when incorporated into polymers. For instance, poly(ethylene terephthalate) or PET is one of those as it has good thermomechanical and barrier properties. Replacing petrochemically derived aromatic monomers can be achieved by using lignin as a source of aromatic constituents. Lignin depolymerization, the process to obtain low-molecular-weight compounds from lignin is a widely explored field [17,38]. Phenolic LMCs such as vanillin, guaiacols, catechols, and cresols afforded by lignin depolymerization supports the prospect of polymerizable LMCs. For example, vanillin is reported to be commercially manufactured from lignin via an oxidation method on a scale of at least 17,000 ton/year [39]. Hence, vanillin has gain significant interest as a monomer precursor for polymer synthesis [40]. Several excellent review articles on lignin-derived aromatic monomer design and lignin polymers are available [41,42]. Compounds such as ferulic acid, vanillin, divanillin, hydroxybenzaldehyde, syringaldehyde and guaiacol are found to be useful starting chemicals towards novel biobased polymers approach (Figure 8).

Polymerization routes, including polycondensation, ADMET, thiol-ene, and radical methods have generated a copious number of lignin-based polymeric materials with a wide window of properties. In this section, ADMET and radical polymerization routes including FRP and RAFT that use monomers derived from lignin or lignin model compounds will be highlighted.

#### 5.3.1. Radical Polymerization Routes (FRP and RAFT)

**Figure 9:**
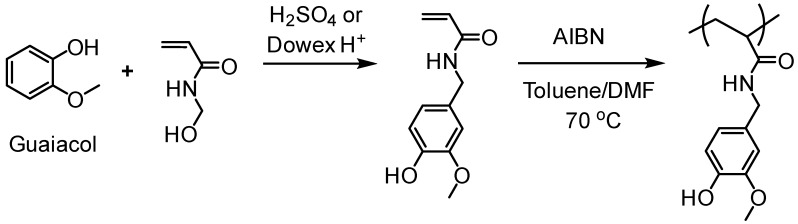
A guaiacol-based acrylamide monomer and polymers by FRP [43].

Natural phenolic compounds such as catechol, eugenol and others represent antimicrobial phytochemicals with useful chemical functionalities for further developments. In 2011, Liu and Roger et al. designed a new generation of antimicrobial polymers based on a natural biocide molecule guaiacol (2-methoxyphenol), obtained from beechwood guaiac resin or wood creosote [43]. Although conventional free radical polymerization (FRP) was used in this work, it is worth highlighting the early developments of the LMC to polymer concept. They used Friedel-Crafts reaction between guaiacol and *N*-hydroxymethyl acrylamide or via three-step synthesis from vanillin to incorporate an acrylamide into the guaiacol architecture (Figure 9). FRPs were carried out using AIBN as the initiator, resulting in polymers with molecular weight (*M*_n_) in the range of 3000–11,000 g/mol and dispersity (*Ð*) of 1.3–1.8.

**Figure 10:**
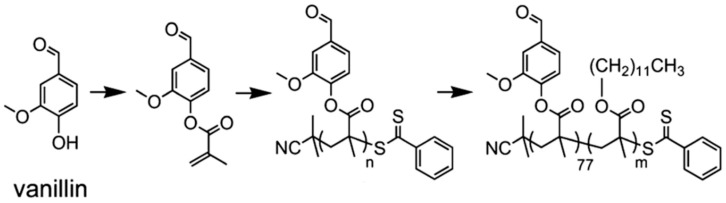
Renewable homopolymers and block copolymers synthesis via RAFT polymerization using vanillin-based methacrylate monomers. Reproduced with permission [44].

Epps and coworkers have carried out extensive work on lignin polymers. In their early investigations, they reported an approach for the synthesis of renewable homopolymers and block copolymers using vanillin, a lignin model compound [44]. They utilized RAFT polymerization to produce well-defined polymers from methacrylate functionalized vanillin (Figure 10). The vanillin-based homopolymers exhibited high glass transition temperatures (120 °C) and thermal degradation temperatures (≥300 °C). In addition, a vanillin-based homopolymer was chain-extended with lauryl methacrylate to produce block copolymers that generated nanostructured materials.

In another study, by Epps et al., bio-oil methacrylate monomers from minimally processed bio-oils, such as pyrolyzed Kraft lignin and vegetable oils, were polymerized to investigate the consequences of structural diversity on the kinetics of RAFT polymerization [45]. This study uncovered two key strategies that improve the viability of bio-based polymers, which minimize the separation costs by polymerizing bio-oil mixtures and prevent batch-to-batch inconsistencies in polymer properties.

The same group reported the synthesis of softwood lignin-based methacrylate monomers, polymerized them via RAFT polymerization and studied thermal and viscoelastic properties [46]. The resultant softwood lignin-based methacrylate polymers possessed excellent glass transition temperatures, thermal stabilities greater than 100 °C above the glass transition temperature, and intermediate shear flow resistances, in comparison to polystyrene and poly(methyl methacrylate).

An investigation into the synthesis of syringyl methacrylate from syringol and its RAFT polymerization was reported [47]. The syringol is a copious component of depolymerized hardwood and graminaceous lignin which makes syringyl methacrylate an excellent monomer to develop biomass-derived polymers. Homopolymers and heteropolymers synthesized from syringyl methacrylate showed good thermal stabilities with broadly tunable and highly controllable glass transition temperatures ranging from 114 to 205 °C. Also, these polymers showed zero-shear viscosities ranging from ∼0.2 to ∼17,000 kPas at 220 °C, demonstrating a wide range of thermomechanical properties that indicate syringyl methacrylate could be a powerful add-in monomer for adjusting materials properties.

Epps et al. reported the effect of para and ortho functional groups on the material properties such as surface energies, solvent compatibilities and friction coefficients of lignin inspired polymer films for coating applications [48]. A series of polymers were generated from methacrylate-functionalized lignin pyrolysis products via RAFT polymerization. The polymer compatibilities with organic solvents increased with increasing aliphatic content in the para position and decreased with the introduction of methoxy groups ortho to the polymer backbone. Also, it was shown that changes in polar moieties, such as aldehydes and methoxies, have greater effects on solubility, surface energy, and friction than changes in the aliphatic groups in the resultant polymers.

**Figure 11:**
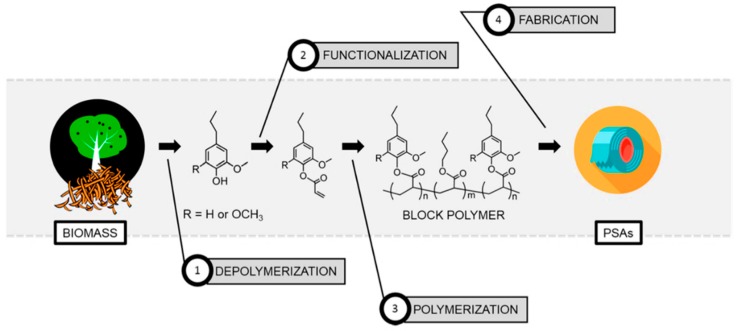
Material development from raw biomass to pressure-sensitive adhesives. Reproduced with permission [49].

High-performance pressure-sensitive adhesives (PSA) developed from lignocellulose biomass were reported by Wang, Epps, and coworkers [49]. In this study, 4-propylsyringol and 4-propylguaiacol were extracted in high purity and yield from depolymerized poplar wood. These aromatic compounds were functionalized with either acrylate or methacrylate groups and polymerized via RAFT polymerization as shown in Figure 11. Resultant polymers displayed excellent adhesion properties such as up to 4 N cm^−1^ 180° peel forces and 2.5 N cm^−1^ tack forces without any tackifier or plasticizer.

**Figure 12:**
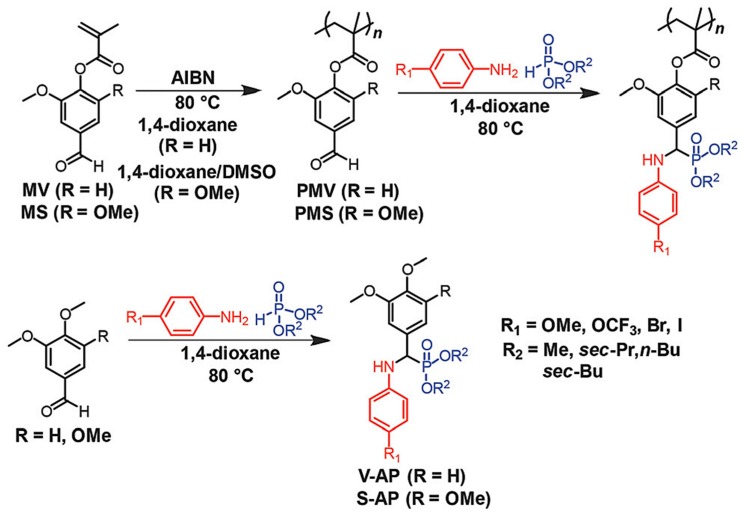
A schematic representation of Kabachnik-Fields post-polymerization modifications of polymethacrylates derived from vanillin and syringaldehyde. Reproduced with permission [50].

A recent study by Maeda et al. reported Kabachnik-Fields three-component reaction (KF-3CR), a type of multi-component reaction (MCR), between amines, phosphites, and lignin-derived polymers featuring vanillin and syringaldehyde [50]. The vanillin and syringaldehyde contain aldehyde groups in their molecular structures which are the most important functional group in MCRs. In this study, polymethacrylates derived from vanillin and syringaldehyde were successfully subjected to KF-PMR (Figure 12). It demonstrated the successful integration of lignin derived chemicals and the MCR-based polymer modification reaction, leading to various functional polymers by taking advantage of the MCRs.

#### 5.3.2. Acyclic Diene Metathesis (ADMET) Polymerization

**Figure 13:**
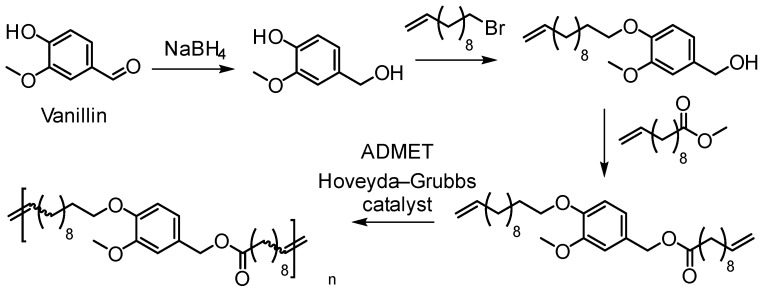
ADMET polymerization of dienes derived from vanillin [51].

Olefin metathesis, including ADMET and ROMP, has become an efficient tool for polymer chemists to produce well-defined polymers [37]. Firdaus and Meier have developed diene monomers from vanillin and fatty acids that were polymerized via ADMET, thiol-ene, and polycondensation type polymerization routes (Figure 13) [51]. ADMET polymerizations led to high molecular weights up to 50,000 g/mol yielding materials with thermoplastic properties. Melting points were in the range of 16 to 78 °C and *T*_g_’s in the range from −37 to −14 °C.

**Figure 14:**
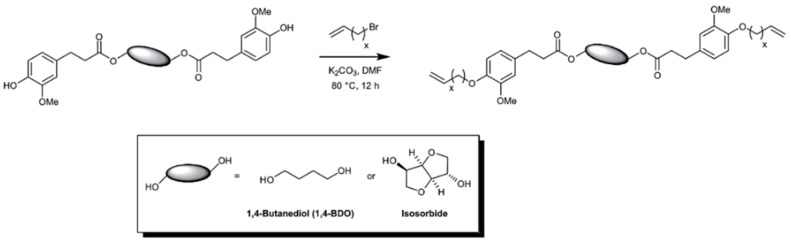
Ferulic acid-derived α,ω-diene monomers. Reproduced with permission [52].

Ferulic acid having the α,β-unsaturated carboxylic acid and phenol functional groups offers a wide range of chemical transformations. Barbara, Allais, and coworkers utilized ferulic acid, bio-sourced diols (isosorbide and butanediol) and bromoalkenes for the synthesis of a new class of biobased polyfunctional molecules (Figure 14) [52]. These novel monomers were polymerized via ADMET polymerization using Hoveyda-Grubbs II catalyst. These new poly(ester-alkenamer)s showed excellent thermal stabilities (283–370 °C) and tunable *T*_g_’s. The same group reported the ADMET polymerization involving sinapic acid derivatives (i.e., syringaresinol, syringaldehyde) as diene substrates [53].

**Figure 15:**
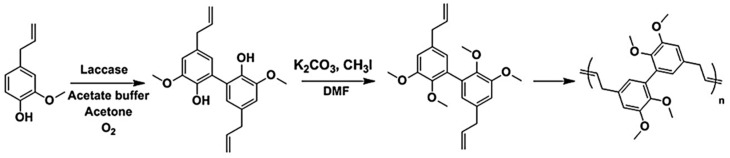
Divinyl compound synthesis from divanillin and ADMET polymerization. Reproduced with permission [54].

Llevot and Cramail et al. reported polyesters, polyethers and conjugated polymers based on divanillin derived monomer (Figure 15) [54]. Starting from 2-methoxy-4-methylphenol, methyl vanillate, vanillin, and eugenol as the substrates, enzymatic dimerization, transesterification, Wittig reaction or allylation led to α,ω-diene monomers. Employing the Hoveyda Grubbs 2nd generation catalyst, the authors were able to generate polymers with molar mass as high as 40,000 g/mol. Polarclean solvent was used as a sustainable, high boiling point and compatible solvent for the polymerization reactions. Due to the rigid backbone, the thermomechanical properties remained high for the conjugated polymers. For example, the *T*_g_ was observed at around 160 °C by DSC and 5 wt % loss temperature occurred at 380 °C. Only trans configuration of the vinylene bonds was observed.

**Figure 16:**
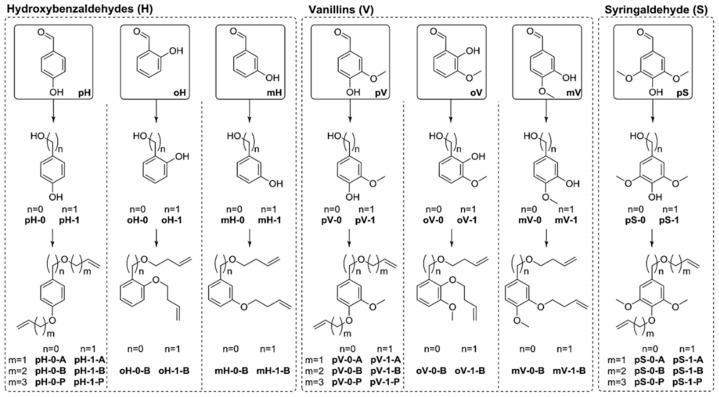
A library of 26 α,ω-dienes with lignin-inspired structural motifs and their ADMET polymerization. Reproduced with permission [55].

Vlaminck, Du Prez and coworkers synthesized a library of 26 α,ω-dienes with lignin inspired structural motifs via Williamson ether synthesis from aromatic diols (Figure 16) [55]. The butenyl and pentenyl monomers resulted in molecular weights between 2500 and 6700 g/mol while allylic monomers showed lower *M*_n_. The polymers had *T*_g_’s ranging from −44 to 18 °C; values could be further increased to 110 °C by efficient post-modification with 1,2,4-triazolinedione (TAD) click chemistry.

### 5.4. Lignin Graft Copolymers

#### 5.4.1. “Grafting Through” Method

**Figure 17:**
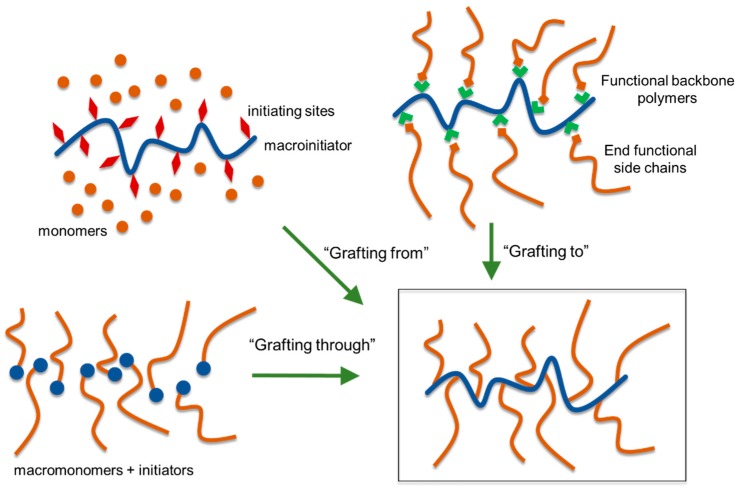
Polymer grafting strategies.

Graft copolymerization provides avenues to combine advantages of physical and chemical properties of both natural lignin and synthetic polymers [56]. Graft copolymers generally consist of a linear backbone of one composition and randomly distributed side chains of a different composition connected to the backbone via covalent bonds. Generally, well-defined graft copolymers can be prepared via (a) a “grafting through” process or (b) a “grafting from” controlled polymerization process or (c) a “grafting to” process such as “click chemistry” (Figure 17) [57].

The “grafting through” approach consists of copolymerization of suitably functionalized lignin with another comonomer(s). The most commonly utilized approach for lignin-based polymer development is the “grafting from” method, in which new polymer chains are directly grown from the initiating sites attached to lignin. This procedure offers a major advantage of high grafting density due to convenient access to monomers to growing polymer chains. A premade polymer with end functionalization is covalently bound to the reactive functional groups of lignin in the “grafting to” method. Although the “grafting to” method offers an advantage to use many types of off the shelf polymers to be attached to lignin, it suffers from low grafting densities resulting from the steric hindrance toward the polymer-polymer reaction.

The grafting of lignin with designer polymers of synthetic or natural sources offers the potential of developing a new class of engineering plastics. At the advent of controlled polymerization techniques, a great interest in advancing lignin-based polymers has arisen. A variety of polymerization techniques have been employed to prepare lignin graft polymers. These methods can be classified into the following groups: (a) free radical polymerization, (b) controlled radical polymerization, (c) ring-opening polymerization, and (d) ring-opening metathesis polymerization. These methods will be discussed in the following sections.

#### 5.4.2. “Grafting From” Method

**Figure 18:**
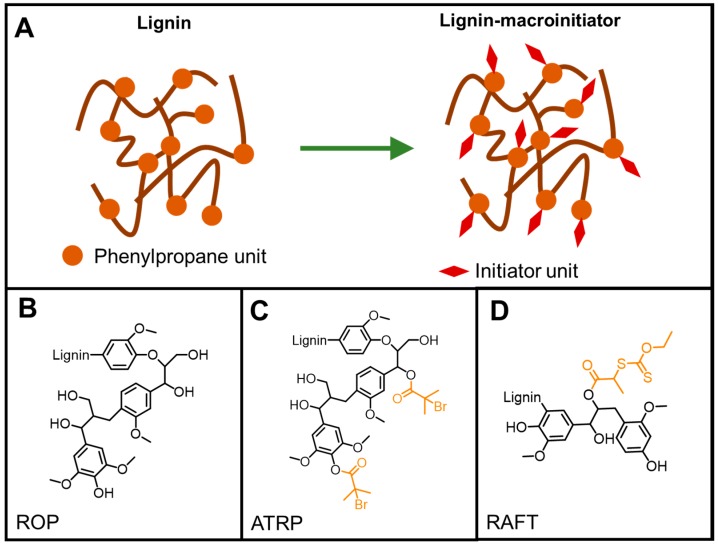
Commonly used lignin-based macroinitiators for controlled polymerizations. (**A**) strategies to prepare macroinitiators; (**B**) ROP macroinitiator; (**C**) BiBB functionalized lignin as macroinitiators for ATRP; (**D**) xanthate functionalized lignin for RAFT polymerization.

Termed as macroinitiators, reactive macromolecules enable a “grafting from” method for polymer synthesis (Figure 18). The rich chemistry of lignin affords initiating site or synthons for initiator attachment applicable to several controlled polymerization methods. While the hydroxy functional groups in lignin work as initiation sites in ROP, a variety of post-polymerization reactions can be employed to covalently attach initiating units on lignin for ATRP, RAFT and ROMP reactions.

#### 5.4.3. Free Radical Graft Polymers

Traditionally, grafting of polymers on to lignin involves the radical polymerization initiated by free radical reactions (chemical or radiation) of an appropriate monomer. The literature on this topic can be found as early as 1968 by Koshijima and Muraki. They used gamma ray irradiation to graft styrene onto hydrochloric acid lignin [58]. Since then, many other reports have appeared using monomers such as styrene, acrylic acid, acrylamides, and vinyl ethers to graft onto lignin via free radical methods. It should be noted that lignin has free radical-scavenging properties as well [59].

The enzyme laccase in the presence of organic peroxides initiated free-radical copolymerization of acrylamide and lignin as observed by Mai et al. [60]. Particularly, dioxane peroxide, tetrahydrofuran peroxide, and t-butylhydroperoxide appeared to be effective in this regard, while H_2_O_2_ showed no such effect. The proposed mechanism of this chemoenzymatically induced graft copolymerization involves the polymerization initiation by peroxy radicals or reduced alkoxy radicals simultaneously with phenoxy radical production. Mai et al. suggested that the poor reactivity of the phenoxy radicals generated by laccase catalysis is not sufficient to start the side chain polymerization. Instead, the covalent bond forming grafting may occur via a termination reaction of the homopolymers initiated by peroxy radicals. The active polymer chain end radical may combine with a phenoxy radical of the lignin backbone [61].

**Figure 19:**
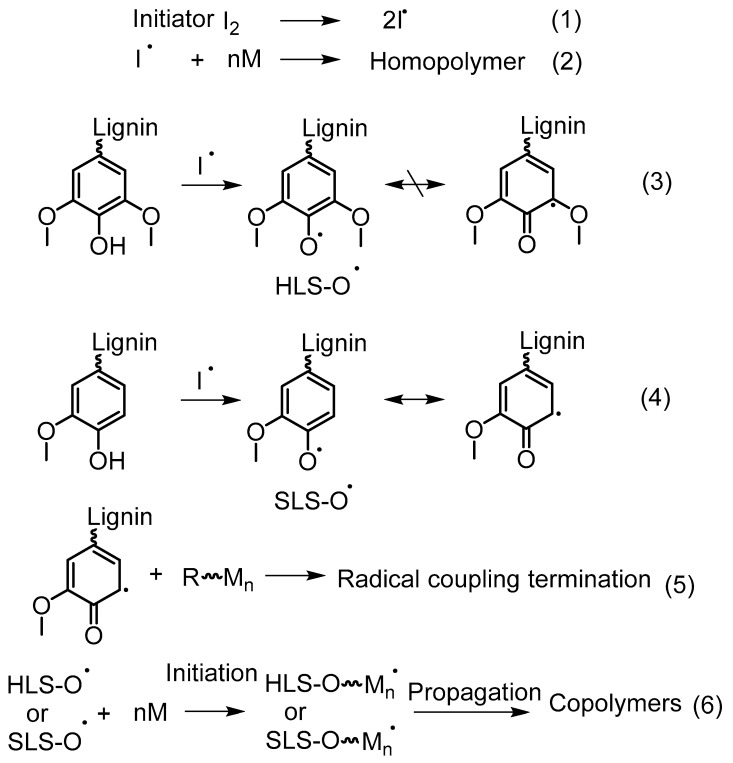
Proposed free radical grafting mechanism of lignin with vinyl monomers. Reproduced with permission [62].

In the recent literature, redox initiation systems are widely used in aqueous graft copolymerization of lignin. For example, CaCl_2_/H_2_O_2_ and K_2_S_2_O_8_ can be given. Ye, Zhang, and coworkers conducted an investigation to explore the mechanism of acrylic acid graft copolymerization on hardwood lignosulfonates (HLS) and softwood lignosulfonates (SLS) [62]. It was evident that the Ph–OH decreased after treatment of K_2_S_2_O_8_, and further after the grafting reaction. Based on this observation, they suggested that Ph–OH acts as a grafting site as well as having negative effects from initiators such as oxidation or radical coupling. The polymerization of acrylic acid was accelerated by the presence of both HLS and SLS compared to acrylic acid homopolymerization. In addition, HLS had a significantly higher contribution compared to SLS. The quinoid radicals in syringly units formed by the self-conjugation of phenoxy radicals may not terminate with active polymer chains due to the steric hindrance caused by the two methoxyl groups. Such radical termination may be more prevalent in the presence of SLS which contain a higher amount of guaiacyl units (Reaction 4 and 5, Figure 19).

Based on these observations, they proposed a possible grafting mechanism of lignin with vinyl monomers (Figure 19). In this mechanism, grafting of the monomers could be resulted by the radical termination between quinonoid structures and growing homopolymer chains, as well as chain initiation by phenoxy radicals. In addition, Ph–OH group may not only participate in the grafting reaction but also with the radical coupling reaction between benzyl and phenoxy radicals as suggested by the dropping content of it after the treatment of initiator.

The use of free radical graft polymerization on lignin novel materials has been carried out in various applications, such as in composites [63], cationic flocculants [64,65], binder in lithium-ion batteries [66], heavy metal ion biosorption [67], anticancer [68], UV-absorbent films [69], and corrosion inhibition [70].

#### 5.4.4. ATRP

ATRP is a popular method to produce graft copolymers, as it is one of the most robust controlled radical polymerization (CRP) methods available. ATRP provides well-defined (co)polymers with predetermined molecular weight, narrow molecular weight distribution, controlled functionalities, topologies, compositions, and a high degree of chain end functionality [71]. Table 2 summarizes the recent efforts in using ATRP to produce lignin graft copolymers.

**Figure 20:**
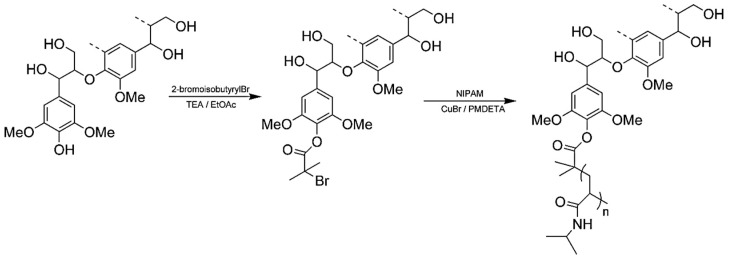
Preparation of lignin–*g*–polyNIPAM copolymers. Reproduced with permission [72].

Generally, ATRP macroinitiators are produced by esterification of lignin hydroxyl groups with 2-bromoisobutyryl bromide. Copolymerization of *N*-isopropylacrylamide (NIPAM) with technical hardwood Kraft lignin via ATRP was reported by Kadla and coworkers (Figure 20) [72]. In this seminal work, they preferentially modified phenolic hydroxyl groups using 2-bromoisobutyryl bromide and triethylamine in EtOAc. The copolymerization reactions were performed at 50 °C using Cu(I)Br/PMDTA as the catalyst system in water/DMF solvent mixture.

The resulting molecular mass of the lignin–*g*–polyNIPAM copolymers was as high as 215,300 g/mol when a moderately high number of initiator sites were available. In the case of the fully substituted macroinitiator, the resulting graft copolymer quickly precipitated out of the solution limiting the polymerization process. These thermoresponsive lignin-based copolymers exhibited a thermally activated phase transition above ∼32 °C, similar to low critical solution temperature (LCST) of polyNIPAM. The thermal decomposition temperature of the lignin–*g*–polyNIPAM copolymers significantly increased with increasing chain lengths.

In a continuation of their work, Kalda et al. immobilized PNIPAM polymer brushes onto the surface of electrospun lignin nanofiber mats by surface-initiated ATRP (SI-ATRP) under aqueous conditions [75]. Softwood Kraft lignin was used to make blends with PEO at concentrations 30 and 0.2 wt % for electrospinning. Chemical crosslinking via oxidative thermostabilization at 250 °C was carried out to improve mechanical performance. Lignin nanofiber mats were treated with different molar ratios of acetyl chloride: 2-chloropropionyl chloride to generate ATRP initiator sites. Aqueous SI-ATRP of NIPAM was carried out using CuCl/HMTETA at room temperature. The lignin graft PNIPAM brushes exhibited ionic responsive characteristics, expanding in water and contracting in a 0.5 M Na_2_SO_4_ aqueous solution.

**Figure 21:**
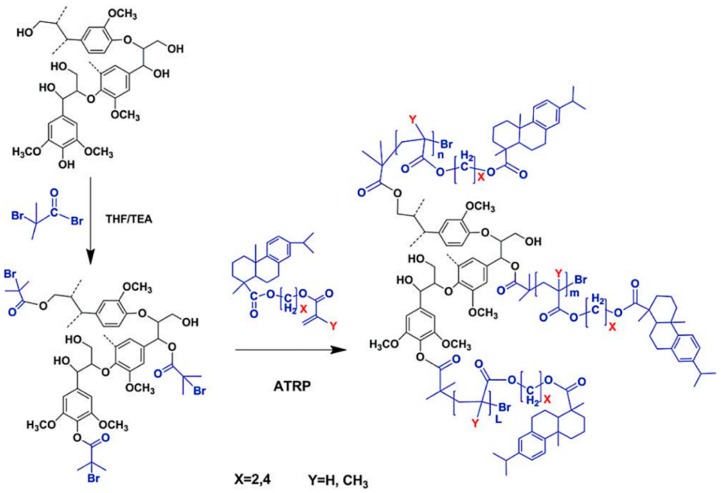
Preparation of rosin polymer-grafted hydrophobic lignin composites. Reproduced with permission [73].

Tang et al. developed several lignin-based biopolymers using a variety of methods. They synthesized rosin polymer–grafted lignin composites via “grafting from” ATRP with the aid of 2-bromoisobutyryl ester-modified lignin as macroinitiators (Figure 21) [73]. The 2-bromoisobutyryl initiating sites were attached to lignin through a simple esterification reaction resulting in lignin ATRP macroinitiators. Three rosin-derived vinyl monomers were sourced for grafting polymers from the lignin macroinitiators. The *T*_g_’s of rosin polymer-grafted lignin composites ranged from ~20 to 100 °C while the contact angle measurements indicated a high hydrophobicity level of the novel materials.

In a more recent work, Tang et al. developed lignin–graft–poly(methyl methacrylate–*co*–butyl acrylate) copolymers via “grafting from” ATRP [74]. These biobased polymers were evaluated as sustainable thermoplastic elastomers. The copolymers exhibited tunable glass transition temperatures and higher thermal stability than unmodified technical lignin. Potential UV-absorbent TPE materials applications of lignins are proposed in this investigation.

Washburn et al. demonstrated the potential of ATRP as the basis for the “one component” composite approach towards more sustainable lignin materials [76]. They made ATRP macroinitiators using ethyl 2-bromoisobutyrate and polymerized styrene or methyl methacrylate using CuCl /BPY in DMF. Mass fraction of lignin varied between 4.5% up to 22.1% and the average degree of polymerization ranged between 323 and 449. The graft copolymers dissolved readily in pyridine or DMF. TEM images revealed a uniform dispersion of lignin particles in PMMA matrix. They observed an increase of *T*_g_ as compared to the homopolymer systems indicating the restriction of grafter polymer chains, particularly pronounced in lignin–*g*–PMMA samples. In addition, higher lignin content resulted in higher moduli suggesting the dependency of composite properties on the intrinsic lignin properties. Compared to binary lignin/polymer blends, polymer-grafted lignin exhibited significantly enhanced fracture toughness, for example, lignin–*g*–PMMA systems had more than 10 times greater toughness. This approach provides a great solution for the poor interfacial binding between filler particles and the matrix of lignin/polymer blends. In more recent work, the same group investigated the properties of PMMA-tethered kraft-lignin particles dispersed in a PMMA matrix [77]. A minute filler content of 1% dramatically enhanced the mechanical properties; a 3-fold increase in yield stress, a 4-fold increase in tensile strength, and a 7-fold increase in toughness.

**Figure 22:**
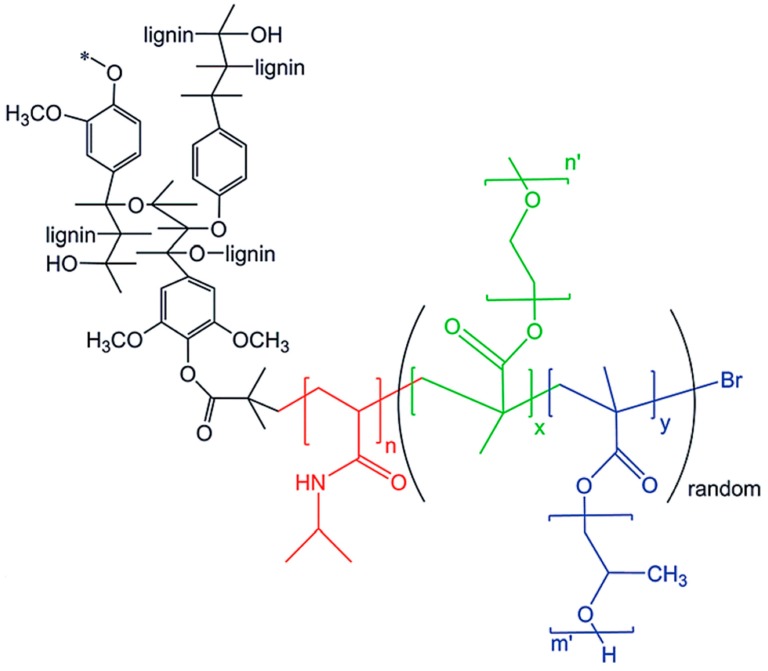
Lignin-based thermogelling graft copolymers. Reproduced with permission [78].

A series of lignin-based thermogelling graft copolymers were developed by Li and coworkers [78]. These materials consisted of a lignin core and multiple arms of graft polymer chains, where each graft consists of a block of poly(*N*-isopropylacrylamide) (PNIPAAm) and a block of brush-like random copolymer of poly(ethylene glycol) (PEG) and poly(propylene glycol) (PPG) (Figure 22). According to the design criteria, lignin core is hydrophobic, the PEG segments are hydrophilic, and PPG/NIPAAM segments are temperature-responsive.

Different degrees of substituted 2-bromoisobutyryl bromide (BiBB) functionalized lignins served as the macroinitiators. A two-step ATRP reaction protocol was followed to produce the graft copolymers. First, NIPAM monomer was polymerized using Cu(I)Br/HMTETA system in 1,4-dioxane at 60 °C for 3 h. The polymer was isolated and used as the macroinitiator for the second step where a mixture of PEGMEMA and PPGMA was polymerized using the same catalyst system at 70 °C for 24 h. These lignin graft copolymers were water-soluble at room temperature and could form aggregations at elevated temperatures turning from sol at ~32–34 °C to hydrogel at a higher temperature. The *G*′ of the hydrogels could be tuned in the range of 2700–13,900 Pa by altering the grafting density by the proper use of lignin-based macroinitiators.

Stimulus-responsive lignin graft copolymers that respond to CO_2_/N_2_ have been developed by Zhu et al. [82]. The objective of their work was to modify alkali lignin by grafting CO_2_-responsive 2-(diethylamino)ethyl methacrylate (DEAEMA) and demonstrate CO_2_/N_2_ switchable dispersion/precipitation of the modified lignin materials in aqueous media. Fully or partially BiBB functionalized lignin was used as the macroinitiator along with CuBr/PMDTA in DMF at 70 °C for the ATRP polymerization of DEAEMA with targeted chain lengths of 1 to 10 DEAEMA repeat units. It was observed that the initiator efficiency of the fully substituted lignin-Br was a little higher than that of the partially substituted lignin-Br.

Lignin–*g*–DEAEMA samples with longer DEAEMA chain lengths took a longer time for flocculation and precipitation whereas shorter one precipitated instantaneously. Homogenizing decane in water with the added lignin samples produced stable Pickering emulsions from N_2_ treated flocculated/precipitated dispersed particles and bubbling CO_2_ could demulsify the emulsion.

**Figure 23:**
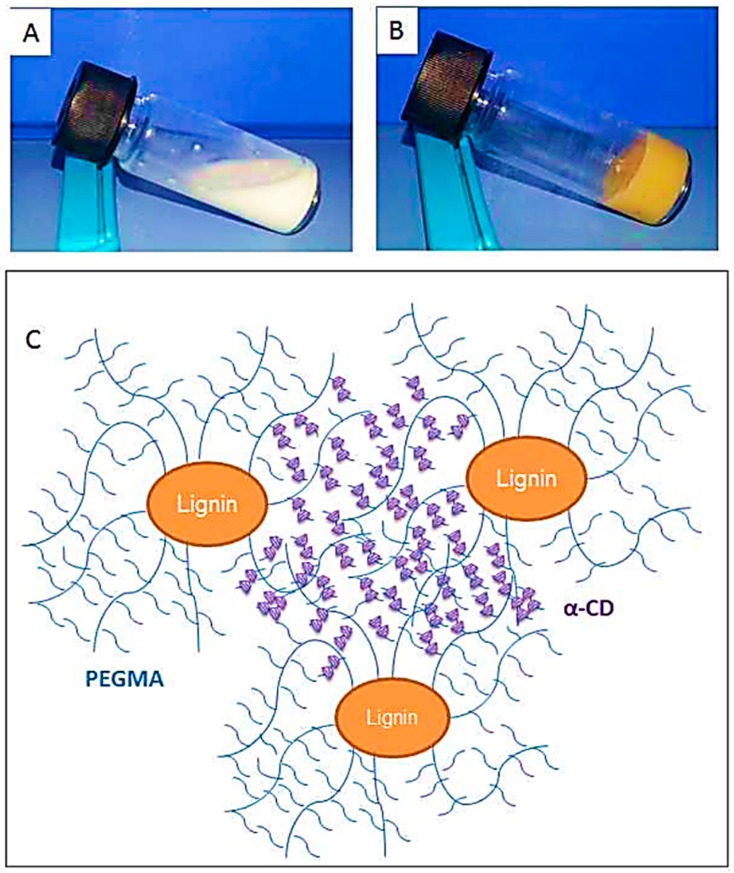
Lignin-based supramolecular hydrogel by inclusion complexation between PEGMA-grafted lignin and α-CD. Reproduced with permission [79].

Kai, Loh, and co-workers developed lignin supramolecular hydrogels with mechanically responsive and self-healing properties. A series of poly(ethylene glycol) methyl ether methacrylate (PEGMA)-grafted lignin hyperbranched copolymers were synthesized via ATRP [79]. The graft copolymers were prepared in a range of molecular weights from 38.7 to 65.0 kDa, where the lignin content ranged from 7.7 to 12.9 wt %. Lignin-Br macroinitiators were prepared using BiBB and the ATRP of PEGMA was conducted using CuBr and HMTETA in acetone at room temperature. In the presence of α-cyclodextrin(α-CD) the aqueous solutions of the graft copolymers were found to form supramolecular hydrogels with a very low critical gelation concentration of 1 wt % copolymers (Figure 23). These hydrogel systems showed mechanically responsive rheological properties and excellent self-healing capabilities where they turned into sol under 10% strain and recovered rapidly (5 s) to the solid state under 0.01% strain. All the lignin–*g*–PEGMA copolymers exhibited excellent cell viability evident from MTT assays using HDF cells. Potentially biodegradable and biocompatible lignin–*g*–PEGMA graft copolymers may have applications in biomedical and personal care fields.

**Figure 24:**
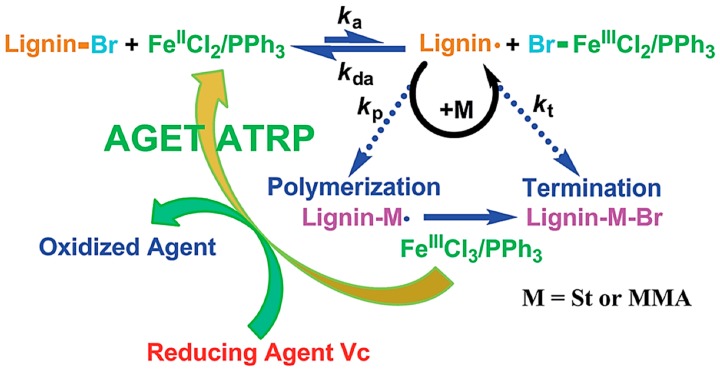
Proposed mechanism for AGET ATRP of lignin graft copolymers. Reproduced with permission [80].

Matyjaszewski et al. developed AGET ATRP (Activators Generated by Electron Transfer) initiation systems that allow the controlled polymerization without the direct involvement of organic radicals or formation of molecules that can act as initiators [83]. A reducing agent is used for the activation of an oxidatively stable catalyst complex. A truly scalable miniemulsion polymerization can be conducted using AGET ATRP demonstrating its industrial applicability [84]. A novel Fe(III)-catalyzed AGET ATRP was used by Wang and co-workers to carry out the graft copolymerization of lignin with styrene and methyl methacrylate [80]. They used FeCl_3_∙6H_2_O as the catalyst, triphenyl phosphine (PPh_3_) as the ligand and ascorbic acid as the reducing agent. A proposed mechanism is illustrated in Figure 24. Environmentally friendly and low-cost iron catalyst seems attractive for lignin-based polymer synthesis.

Zhang, Li, and coworkers modified kraft lignin by grafting from 2-(dimethylamino)ethyl methacrylate (DMAEMA) starting from the lignin-based macroinitiators esterified with esterification with BiBB [81]. These graft copolymers having hydrophilic and cationic polymer chains were able to bind to pDNA and form polyplex nanoparticles with size ranging from 100 to 200 nm at *N*/*P* ratios of 5 or higher. Luciferase assay indicated that graft copolymers with very short arm lengths (average DP 5.5) show in vitro transfection efficiency comparable to or higher than branched PEI. The cytotoxicity of lignin–*g*–PDMAEMA copolymers increased with increasing PDMAEMA arm length.

##### RAFT Polymerization

RAFT polymerization is a reversible-deactivation radical polymerization method that uses a chain transfer agent (RAFT agent, CTA). Lignin polymeric advancements in using RAFT polymerization route for the “graft from” polymers are summarized in Table 3.

Washburn et al. prepared lignin-based surfactants using polyacrylamide and poly(acrylic acid) that were “graft from” lignin macroinitiators via the RAFT polymerization method [85]. In the synthesis route, potassium xanthate was reacted with 2-bromopropionic acid to produce xanthate carboxylic acid, which was subsequently esterified with lignin. The RAFT polymerization was carried out in DMF at 70 °C with acrylamide or acrylic acid monomers and AIBN as the radical initiator. To analyze the graft copolymer, it was cleaved from the lignin core using KOH hydrolysis. It was estimated that the grafting densities obtained were approximately 2 and 17 polymers per lignin particle based on the low and high CTA grafted macroinitiators respectively. In one instance, polyacrylamide with *M*_n_ of 11,300 g/mol and Ð of 1.83 was obtained. All polymer-grafted lignin compositions were soluble in water at a concentration of 1 mg/mL and were surface active, reducing the surface tension to as low as 60 dyn/cm. They proposed that these lignin graft copolymers form random patchy nanoparticles with mixed hydrophilic and hydrophobic domains that result in unexpected interfacial behaviors. In a later report, Washburn and co-workers investigated the properties of Pickering emulsions produced using lignin–*g*–polyacrylamide nanoparticles [86].

**Figure 25:**
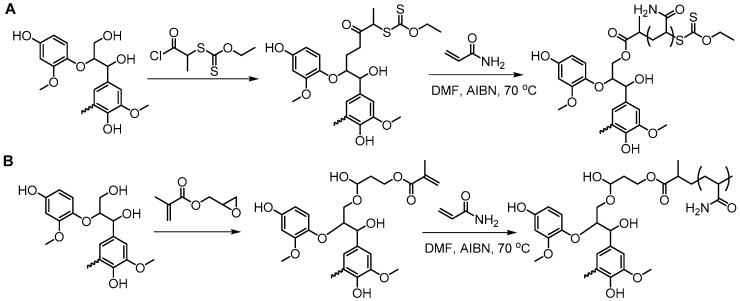
Synthesis of lignin–*g*–polyacrylamide via (**A**) RAFT and (**B**) FRP. Reproduced with permission [88].

A class of anionic polymer dispersants called superplasticizers are employed to inhibit aggregation in and fusion of hydraulic cement. This helps to reduce the yield stress of cement paste, lower the water requirements and extend the workability of cement. Molecular architecture requirements for polymer-grafted lignin superplasticizers was investigated by Gupta and Washburn et al. They developed high-performance superplasticizers made of polyacrylamide-grafted lignin prepared via RAFT and free radical polymerization (FRP) (Figure 25) [88]. The results indicated that high-performance dispersants are obtained through a grafted architecture using CRP of acrylamide using RAFT, whereas FRP resulted in a nanogel that lacked the dispersant performance.

Kurtis, Washburn and co-workers extended their lignopolymer work to develop effective plasticizers for Portland cement blended with two natural, finely divided minerals kaolin clay and clinoptilolite zeolite [87]. Basically, they studied the plastic behavior of mineral-cement combinations dosed with polyacrylamide-grafted kraft lignin and compared with lignosulfonate and commercial polycarboxylate ether. Lignopolymer was found to adsorb strongly to both kaolin and clinoptilolite and resulted in the lowest yield stresses in pastes indicating effective superplasticizer properties.

Tang et al. developed lignin and soy oil-derived polymeric biocomposites by “grafting from” RAFT polymerization using organosolv lignin as the core [89]. They prepared the lignin-based chain transfer agent (Lignin-RAFT) by simple esterification between 4-cyano-4-(phenylcarbonothioylthio) pentanoic acid and lignin in the presence of DCC and DMAP at room temperature. Then, soybean oil-based methacrylate monomers were polymerized via RAFT polymerization using Lignin-RAFT and AIBN at 70 °C in dry toluene. Secondary amide containing polymers exhibited a higher glass transition temperature due to the formation of hydrogen bonds.

A new kind of cationic flocculant was developed by Liu and coworkers via Steglich esterification of phenolated kraft lignin and subsequent RAFT polymerization with 2-(methacryloyloxy)ethyl trimethyl ammonium chloride to produce multi-arm star copolymers with a lignin core [90]. 2,2′-azobis (2-methylbutyronitrile) was used as the initiator and DMF was used as the solvent. The polymers had a high molecular weight of 10^5^–10^6^ g/mol that is essential for the flocculation applications. The flocculation performance in the removal of kaolin particles from simulated wastewater was evaluated. A maximum removal efficiency of 96.4% within 1 h settling was observed.

##### ROP

Ring-opening polymerization (ROP) is a well-established, industrially practiced form of chain-growth polymerization. Cyclic monomers react with anionic, cationic or radical initiators to produce polymers with functional groups such as ether, ester, amide, and carbonate. Some of the current literature on lignin usage via ROP polymerization for the “graft from” polymers is summarized in Table 4.

**Figure 26:**
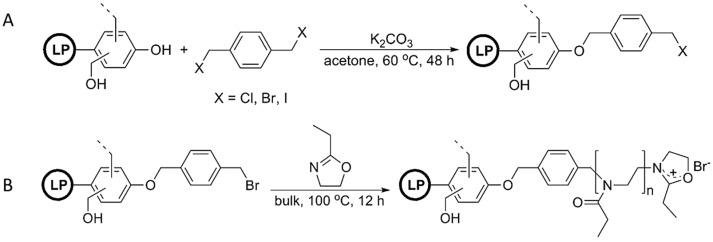
(**A**) Modification of organosoluble lignophenol with α,α′-dihalo-*p*-xylene; (**B**) Cationic ring-opening polymerization of 2-ethyl-2-oxazoline. Reproduced with permission [91].

Polymers derived from the ring-opening polymerization of 2-alkyl-2-oxazoline are miscible with various types of commodity polymers such as poly(vinyl chloride) (PVC), polyvinylpyrrolidone (PVP), polycarbonate (PC), or polystyrene (PSt). Nemoto, Konishi, and co-workers envisioned that lignin-based recyclable natural phenolic resins or high-performance polymer blends can be prepared by grafting such polymers from lignin [91]. As the first step, they modified lignophenol (LP) via Williamson ether synthesis of α,α′-dihalo-*p*-xylene to produce a halomethylphenyl-group-modified lignin, where LP-Br was estimated to be 1.50 mmol/g (Figure 26). Subsequently, the LP-Br was used to initiate the cationic ROP of 2-ethyl-2-oxazoline resulting in “graft from” poly(2-ethyl-2-oxazoline). These lignin graft copolymers clearly indicated the miscibility with PVC and PVP.

Chakraborty, Mandal, and coworkers developed a lignin-based anti-infective ointment to control persistent inflammation [92]. They synthesized lignin–graft–polyoxazoline conjugated triazoles as the new materials. Tosylated lignin macroinitiators were used to conduct the cationic ROP hydrophilic 2-methyl oxazoline monomers. The copolymer was covalently modified with triazole moiety to enhance the antimicrobial and anti-biofilm activities. A lignin loading of 20 wt % was found to be appropriate for hydrogel formation with spherical copolymer nanoparticles of 10–15 nm size. These lignin-based materials have demonstrated abilities to prevent infection of burn wounds, aid healing, and act as an anti-inflammatory dressing material.

**Figure 27:**
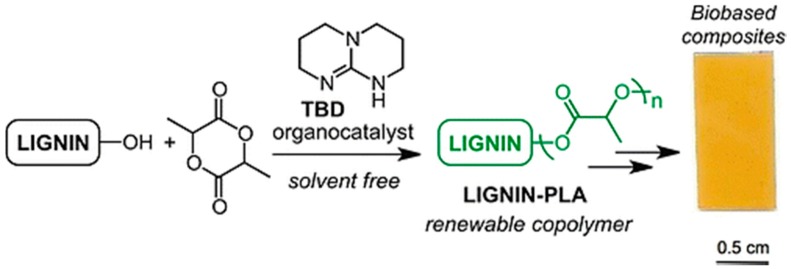
Ring-opening polymerization of lactide on lignin using triazabicyclodecene (TBD) organocatalyst. Reproduced with permission [93].

Chung and Sattely et al. developed a catalytic and solvent-free method for synthesis of a lignin–*g*–poly(lactic acid) (PLA) copolymer to improve the miscibility of lignin with other biopolymers (Figure 27) [93]. In this method, graft polymerization of lactide onto lignin was catalyzed by triazabicyclodecene (TBD). Preacetylation treatment or varying the lignin/lactide ratio demonstrated to be useful handles to control the PLA chain length. It was evident that high grafting efficiency and preferential grafting on lignin aliphatic hydroxyls over phenolic hydroxyls. The graft copolymers displayed *T*_g_’s ranging from 45 to 85 °C and multiphase melting behavior. Incorporation of lignin resulted in an increase in tensile strength and strain while maintaining tensile modulus.

**Figure 28:**
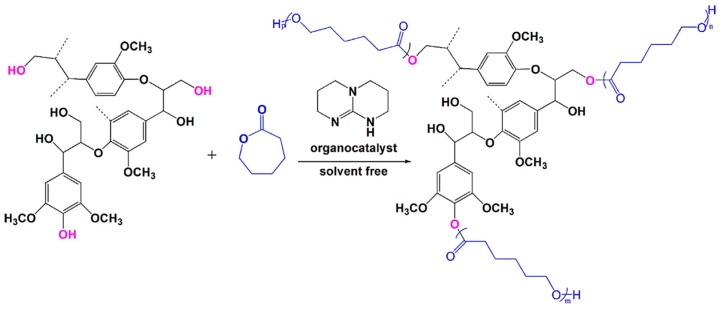
Solvent-free ROP to produce lignin–graft–poly (ε-caprolactone) copolymers. Reproduced with permission [94].

Production of biobutanol from corn or wheat straw results in lignocellulosic butanol residue as a co-product. Liu and coworkers developed a “graft from” ROP technique to fabricate lignin–graft–poly(ε-caprolactone) copolymer (BBL–*g*–PCL) using biobutanol lignin (BBL) as raw material recovered from lignocellulosic butanol residue (Figure 28) [94]. A series of BBL–*g*–PCL copolymers with different molecular weights, ranging from 367 to 8200 g/mol were synthesized. They observed better dispersion and UV-protective properties of these copolymers.

Muller et al. investigated the effects of grafted chain lengths of polycaprolactone and lignin content toward nucleation, crystallization, and thermal fractionation [99]. It was found that the competition between lignin nucleation and PCL–*g*–lignin intermolecular interactions determines the crystallization behavior of the copolymer. At low lignin contents (2–5 wt %), the nucleation of lignin contributes more to the behavior due to the limited intermolecular hydrogen bonds. Higher lignin content induces large antinucleation effects, where hydrogen bond act as physical crosslinks that limit crystallization and lamellar sizes of PCL segments. This resulted in large decreases in both crystallization and melting points.

He et al. fabricated PLA-lignin composites by blending lignin–*g*–rubber–*g*–poly(d-lactide) copolymer particles and commercial poly(l-lactide) (PLLA) [95]. Lignin–*g*–rubber–*g*–poly(d-lactide) copolymer was synthesized by the lignin-initiated ring-opening copolymerization of an ε-caprolactone/l-lactide mixture that resulted in a rubbery layer, followed by the formation of poly(d-lactide) (PDLA) outer segments via the polymerization of d-lactide. They envisioned that the PDLA segments may contribute to strong interfacial interactions between lignin-rubber-PDLA and PLLA matrix by stereocomplexation, as supported by the DSC measurements. More recently, Kai, Loh, and coworkers investigated a series of new lignin-based copolymers lignin-poly(ε-caprolactone–*co*–lactide), lignin-PCLLA for the potential use in healthcare applications [96]. Copolymers with tunable glass transition temperatures (−40 to 40 °C) and molecular weights (10 to 16 kDa) were obtained. Blending the copolymers with polyesters such as (polycaprolactone, and poly(l-lactic acid) was achieved via electrospinning. The ultrafine nanofibers were engineered that exhibited antioxidant activity and biocompatibility. Incorporation of lignin copolymers significantly improved the mechanical properties of PCL nanofibers. However, a negative effect was observed for the PLLA nanofibers.

Polyhydroxyalkanoate (PHA) is a class of commercially available sustainable aliphatic polyesters produced by various microorganisms for energy storage purposes [97]. While there are several types of PHAs, poly(3-hydroxybutyrate) (PHB) has excellent features such as biodegradability, biocompatibility, and thermoplastic behavior. Kai, Loh, and coworkers developed lignin-PHB copolymers to enhance the mechanical properties of PHB. They made nanofibers from these modified lignin polymers via electrospinning. Composites with 2% lignin showed improved tensile strength, elongation as well as tunable antioxidant properties and biocompatibility.

**Figure 29:**
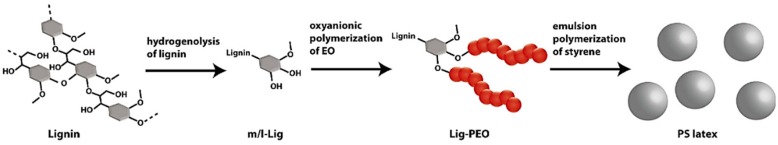
Lignin-based surfactants and their utilization in emulsion polymerization. Reproduced with permission [98].

Oxyanionic polymerization of ethylene oxide initiated by lignin precursors was investigated by Schmidt et al. [98]. They were able to produce non-ionic surfactants with grafting of poly(ethylene oxide) from renewable lignin fragments that were formed by hydrogenolysis. These surfactants were used for the emulsion polymerization of styrene (Figure 29).

##### ROMP

**Figure 30:**
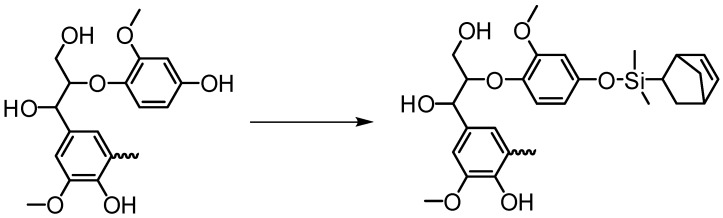
Lignin-based ROMP macroinitiator structure before a ruthenium catalyst is covalently attached [100].

Washburn et al., in their patent disclosure, indicated that ring-opening metathesis polymerization (ROMP) may be used to prepare lignin-based graft polymers using a lignin-derived macroinitiator (Figure 30) [100]. In this approach, a ruthenium catalyst is covalently attached to the lignin surface, and strained monomers such as norbornene may proceed to producing “grafting from” polymers. Although lignin “grafting from” via ROMP is not well explored, there are reports of applicable chemistries where norbornyl group is used to tether Grubbs′ catalyst to surfaces to make macroinitiators followed by controlled polymerization of the monomers [101].

#### 5.4.5. “Grafting To” Method

The availability of modular and orthogonal “click” and related chemistries has enabled the more efficient “grafting to” method for the preparation of graft copolymers. In this method, preformed functional polymers are covalently connected to the substrate via a “click”-type coupling reaction. This allows for precise control and tuning of properties of the resulting material. Click reactions used in macromolecular synthesis is reviewed elsewhere [102,103]. While azide-alkyne cycloaddition reaction is commonly used to make well-defined lignin graft polymers other chemistries such as thiol-based reactions, Diels-Alder, azo coupling, etherification, esterification, and urethanization are also explored.

##### Azide–Alkyne Cycloaddition

**Figure 31:**
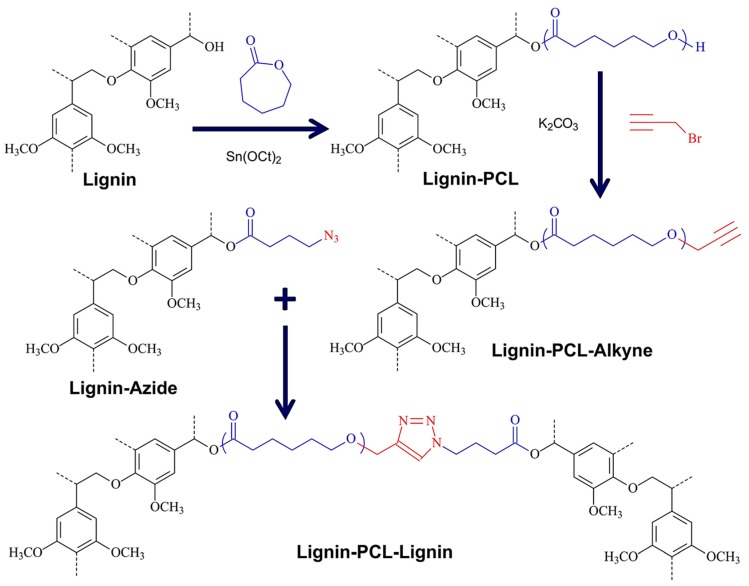
Synthesis of lignin copolymers via copper-free thermal click chemistry of Lignin-PCL-Alkyne with Lignin-Azide functional macromolecules. Reproduced with permission [104].

Tang et al. demonstrated a simple procedure to the preparation of lignin-grafted polymers with tunable compositions including PEG, PCL, and PLA via robust metal-free thermal azide−alkyne cycloaddition reaction (TAAC) [104]. Lignin derivatives with reactive functional groups with either alkyne or azide groups were prepared through facile chemical modifications of lignin hydroxyl groups. Lignin-PCL and Lignin-(PCL–*co*–PLA) copolymers were prepared via ROP and functionalized via esterification reactions. Macromolecules including Lignin-Azide, Lignin-Alkyne, Lignin-PCL-Alkyne, and Lignin-(PCL–*co*–PLA)-Alkyne were paired appropriately and further treated thermally to produce polymer-polymer conjugates. Figure 31 illustrates an example of the Lignin-PCL-Lignin copolymer synthesis route. This approach enables the utilization of biomass toward low-cost scalable renewable polymers and composites.

**Figure 32:**
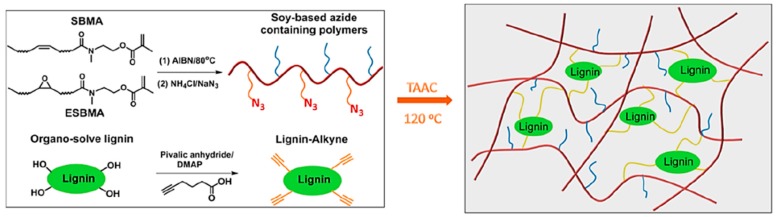
Biobased elastomers prepared by combining soy-based azide-containing polymers and lignin-alkyne. Reproduced with permission [105].

Thermoplastic elastomers (TPE), also referred to as thermoplastic rubbers, are an important type of elastomers that within their purposed limits behave similar to thermoset rubber but are melt processable above their melt or softening temperature. Unlike thermoset materials, TPEs can easily be reprocessed and remolded extending their useful life span. This important feature is typically obtained by incorporating hard domains in a matrix of elastic compositions. In a more recent paper, Tang et al. developed TPEs by combining soybean oil-based azide-containing polymers and alkyne functionalized lignin via TAAC (Figure 32) [105].

The elastomeric matrix made of a soybean oil methacrylate polymer with a *T*_g_ of −6 °C was prepared by free radical polymerization. Subsequently, azide-functionalized polymers were afforded by nucleophilic ring-opening of the oxirane group on the fatty pendant groups by sodium azide. Direct esterification coupling between hydroxyl groups on lignin and 5-hexynoic acid generated the alkyne functionalized lignin. The two types of polymers were dissolved in varying ratios in THF and poured into Teflon molds, solvent removed, and the curing was carried out at 120 °C. The resulting soybean oil-based elastomers exhibited high mechanical strength and excellent elasticity.

**Figure 33:**
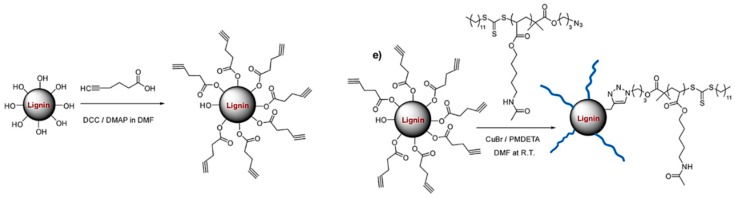
Synthesis of Lignin–graft–poly(5-acetylaminopentyl acrylate). Reproduced with permission [106].

Chung et al. reported a new lignin-containing functional polymer, lignin–graft–poly(5-acetylaminopentyl acrylate) (Lignin–graft–PAA) by the covalent linkage of chemically modified lignin with end-group functionalized polymer PAA [106]. They prepared PAA via RAFT polymerization of the monomer with multiple hydrogen-bonding sites. The azide functionalized RAFT agent provided the chain end functionality to the PAA polymer. Softwood lignin was coupled with 5-hexynoic acid via DCC coupling to afford the alkyne functionality. A sophisticated structural modification was achieved by grafting the polymer to lignin via the copper-catalyzed azide-alkyne cycloaddition reaction (Figure 33). PAA polymer arm lengths of DP 300 and DP 500 were used to create lignin–graft–PAA with a lignin weight percent of 10, 15, 20, and 25 wt %.

Lignin–graft–PAA showed uniform dispersion indicating that lignin and PAA polymer are highly compatible. Young′s modulus, maximum tensile strength, and energy-to-break increased with increasing lignin content. However, at 25 wt% lignin loading, the material became brittle. It was found that such chemical modifications impart autonomic self-healing properties to the lignin-containing polymers.

Westwood and co-workers developed a new method to convert γ-hydroxyl of the β-O-4 unit in a butanol-extracted organosolv lignin into azide groups [107]. Tosylation of the lignin followed by azide substitution enabled the formation of a triazole-bridged connection from lignin to a substrate of choice. 2D HSQC NMR analysis of the products obtained via a thermal protocol between a model alkyne and lignin confirmed the formation of both the 1,4- and 1,5-triazole isomers, as expected. However, regioselective CuAAC variant was preferred due to the formation of only the 1,4-triazole isomer that may result in a more homogeneous material. They were able to develop PEGlylated lignin using the above chemistry, and found that the resulting material exhibit a *T*_m_ of 267 °C, while unmodified lignin had a lower *T*_m_ at 246 °C. The TGA indicated about 14 °C increase of *T*_onset_ of thermal degradation for the new materials. These results provide the evidence for an increase in thermal stability of the PEGlylated lignin.

##### Thiol-Based Reactions

Thiol-based “click” reactions including thiol-ene and thiol-michael addition has gained much attention with regard to macromolecular design [108,109]. There are several reports indicating the use of thiol-ene reactions to graft small functional molecules or polymers to lignin. The reaction can be photochemically or thermally driven. Generally, for photochemical thiol-ene reaction, two methods are available: by photoinitiator and by photoredox catalyst. Zhang and Kong et al. developed *N*-acetyl-l-cysteine functionalized bamboo lignin via UV-initiated thiol-ene reaction for the enhanced adsorption of Cu(II) and Pb(II) [110]. In this work, they isolated lignin from bamboo feedstock using an acetic acid method. The lignin was allylated and coupled with *N*-acetyl-l-cysteine under UV irradiation in the presence of the photoinitiator 2,2-dimethoxy-2-phenylacetophenone (DMPA). Lawoko and co-workers selectively allylated the ethanol-soluble fraction of Lignoboost Kraft lignin using allyl chloride via a mild and industrially scalable approach [111]. Later, they used thermally induced initiator-free thiol-ene chemistry to produce lignin thermosets.

**Figure 34:**
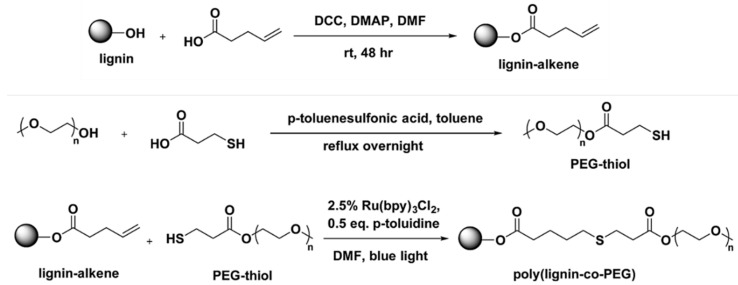
Lignin modification to prepare lignin-alkene and thiol-ene reaction of lignin-alkene and PEG-thiol. Reproduced with permission [112].

The Chung research group reported a low energy and environmentally friendly lignin modification method induced by visible blue light via a photoredox thiol-ene reaction (Figure 34) [112].

They allylated softwood kraft lignin was prepared by DCC coupling of 4-pentenoic acid and lignin. A variety of thiol compounds and PEG-thiol were used for the thiol-ene reaction. Out of three different photochemical reagents, Ru-(bpy)_3_Cl_2_, Eosin Y, and DMPA, the best performing catalyst was found to be Ru-(bpy)_3_Cl_2_. They observed high conversion between 81% and 97% at relatively short reaction times between 60–180 min under low energy 3 W blue LED light or even by 4 h irradiation of natural sunlight.

Habibi and co-workers made fully biobased maleimide-lignin derivatives by first coupling soda lignin and 11-maleimidoundecylenic acid followed by coupling with multifunctional thiol linkers to produce insoluble polymer networks out of the maleimide-lignin at room temperature, under solvent- and catalyst-free conditions [113]. The final lignin content in the new lignin-based polymeric materials was in the range of 30–40%. By varying the linker functionality, the thermal and mechanical properties of the materials could be widely tuned.

##### Diels-Alder Reaction

**Figure 35:**
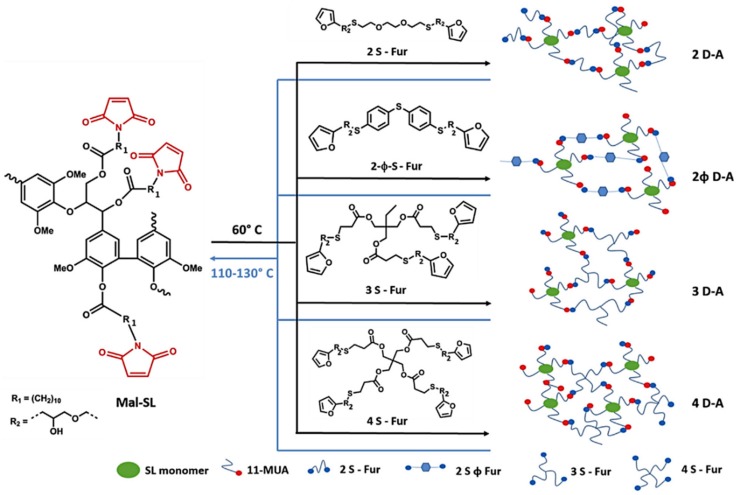
Thermo-reversible D-A polymerization of maleimide grafted lignin with the multifunctional furan linkers. Reproduced with permission [116].

The furan-maleimide Diels-Alder reaction has become a versatile click-unclick tool in macromolecular design to fabricate thermally reversible advanced materials [114,115]. Habibi and co-workers prepared thermo-reversible healable materials based on lignin (Figure 35) [116]. In their approach, soda lignin was esterified with 11-maleimidoundecylenic acid to produce maleimide-containing lignin derivatives that were subsequently homogenized in DCM and polymerized through the D–A click polymerization at 60 °C with different multifunctional furan linkers. The polymeric networks demonstrated self-healing properties when scratched and further cured at 110–130 °C.

##### Azo Coupling Reactions

**Figure 36:**
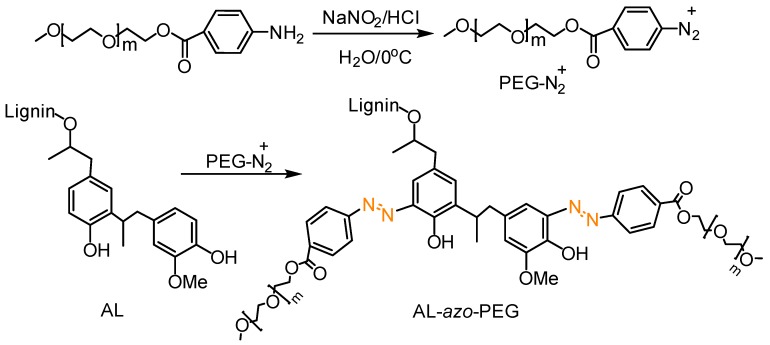
Synthetic route to produce PEGylated alkali lignin (AL-*azo*-PEG). Reproduced with permission [120].

Azo coupling reaction is simply the reaction between a diazonium compound and another aromatic compound that produces an azo compound. It is a useful tool to prepare organic azobenzene molecules or side chain azo polymers with a high degree of functionalization [117,118]. Qiu, Qian and co-workers prepared lignin-based azo polymers from alkali lignin [119]. UV-blocking performance of hollow lignin azo colloids and their controlled release of photosensitive pesticide avermectin was investigated. Macromolecular functionalization via post-*azo*-coupling reaction was efficiently utilized to synthesize advanced lignin grafted polymers (Figure 36) [120]. In this work, He et al. PEGylated alkali lignin to make water-soluble lignin-based polymers by a one-step coupling reaction between lignin and PEG-based macromolecular diazonium salts in alkaline water. Interestingly, the graft polymers had good solubility both in water over a wide pH range (pH 2–12) and in many organic solvents. Self-assembled colloidal particles and nanofibers were developed by vapor diffusion and electrospinning. In addition, photo-responsive properties were observed in these lignin azo polymers.

##### Etherification 

Lignin hydroxyl groups are often etherified or esterified to produce functional materials. Washburn et al. prepared PEGylated lignin to study the effects of PEGylation on the interfacial activities [121]. They modified kraft lignin and sodium lignosulfonate using a simple etherification reaction. First, they mesylated mPEG hydroxyl groups using methanesulfonic anhydride. The lignin phenolic hydrogyl groups were then reacted with mPEG-Ms at high pH conditions via nucleophilic substitution chemistry which generated a grafting density of ca. 5 polymer grafts per lignin core. PEGylated lignin demonstrated modest reductions in air-water surface tension and formulations based on PEGylated kraft lignin are significantly more effective at stabilizing oil-water interfaces than PEGylated lignosulfonate.

Epoxy ring opening reaction has shown utility in grafting small molecules and polymers as well as producing cross-linked lignin thermosets. Lignin-based water-soluble polyoxyethylene ether was synthesized by Qui et al. via covalent grafting epoxy functionalized poly(ethylene glycol) to kraft lignin [122]. Reactive PEG polymers with various lengths were fabricated via chain end functionalization using epichlorohydrin and the catalyst BF_3_-Et_2_O. Then, kraft lignin phenolic hydroxyl groups were blocked by etherification with the PEG-chlorohydrin intermediate under alkaline condition. The lignin-PEG copolymer was used as a dispersant for 50% dimethomorph agricultural suspension concentrates and demonstrated improved dispersing and rheological properties compared to lignosulfonate and PEG.

##### Esterification 

A novel lignin-based targeted polymeric nanoparticles platform was developed by Lei, Wang, and Ma et al. utilizing folic acid-polyethylene glycol-alkaline lignin conjugates [123]. A simple esterification method was followed to covalently attach alkaline lignin and folic acid functionalized PEG.

**Figure 37:**
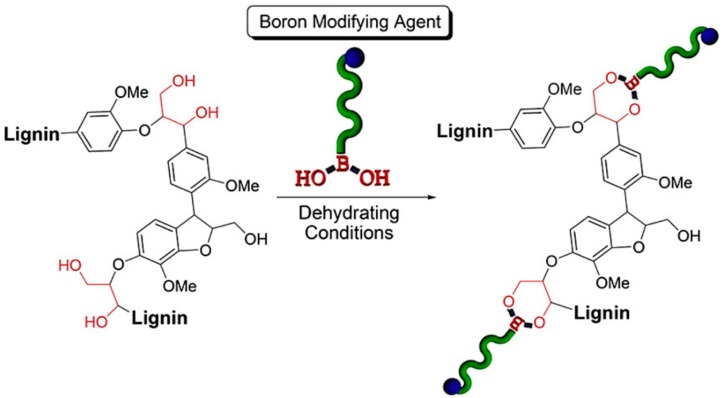
Modification of organosolv lignin using arylboronic acid end-functionalized polymers. Reproduced with permission [126].

Boronic acid-containing macromolecules have gained attention in the polymer and materials science to produce dynamic covalent materials, dual thermo- and saccharide-responsive hydrogels, sensors, and nanomaterials [124]. Arylboronate ester linkages are formed between the reaction of boronic acids and 1,3-diols. In a groundwork investigation, Iovine et al. synthesized arylboronate ester-modified artificial lignins [125]. The presence of 1,3-diols in lignin’s most prevalent structural subunit (β-O-4) indicated the possibility of grafting to lignin using boronic acid chemistry. Iovine et al. more recently developed PCL graft organosolve lignin using a novel “graft to” approach (Figure 37) [126]. The graft copolymers were prepared by covalently linking boron end-functionalized polycaprolactone PCL homopolymers with organosolv lignin via reversible covalent bonds consisting of arylboronate ester bonds.

##### Urethane Linkages

**Figure 38:**
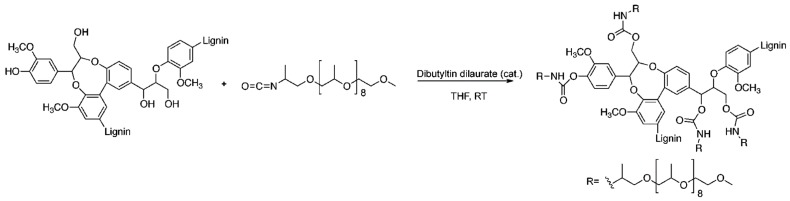
Polyurethane synthesis from lignin and an isocyanate-terminated poly(propylene oxide) macromonomer. Reproduced with permission [128].

The interest in making polyurethanes and polyesters from lignin for applications in adhesives and coatings is immense [127]. Diisocyanates (or polyisocyanates) and polyols with terminal hydroxyl groups react to form polyurethane (carbamate) groups. In terms of the “grafting to” method, urethane chemistry has great potential in lignin chemistry due to the abundance of phenolic and aliphatic hydroxyl functional groups in lignin. For example, Figure 38 illustrates the polyurethane synthesis from organosolv lignin and an isocyanate-terminated poly(propylene oxide) macromonomer catalyzed by dibutyltin dilaurate as reported by Gandini et al. [128].

**Figure 39:**
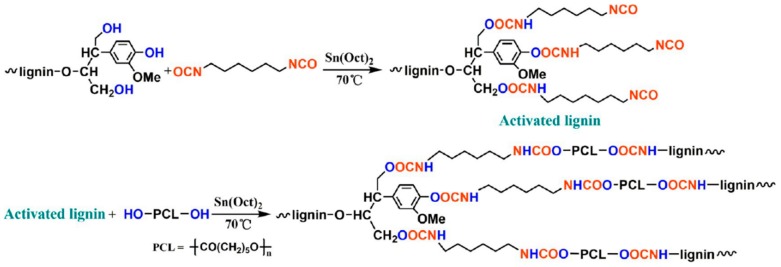
Polyurethane bioplastics derived from lignin-poly(ε-caprolactone). Reproduced with permission [129].

Zhang, Fang, and coworkers reported renewable high-performance polyurethane bioplastics derived from lignin-poly(ε-caprolactone) (Figure 39) [129].

They incorporated poly(ε-caprolactone) as a biodegradable soft segment to lignin using hexamethylene diisocyanate as the reactive grafting agent. The polyurethane film possessed high performance in tensile strength (19.4 MPa), breaking elongation (188%), and tear strength (39 kN/m) when the lignin content was as high as 37%. In addition, the thermal stability of the new materials was excellent compared to purified lignin.

## 6. Conclusions and Future Prospects

Lignin chemistry has fascinated many generations of scientists, scholars and industry leaders. There certainly are many opportunities in developing new materials based on lignin with tailor-made physical properties. The complex and variable molecular architecture of lignin provides a challenging avenue to move forward. Due to the variability of the chemical structure, lignins can be considered as macromolecules with a generally known structure that depends on the plant source and chemical reactions used in isolation. Thus, each family of lignins requires specific characterization before manipulation for targeted end-use applications. As discussed above, controlled polymerization methods such as ATRP, RAFT, and ADMET, as well as polymer “grafting to” chemistries such as versatile “click” reactions, have facilitated many exciting innovations leading to a variety of chemical structures, physical properties, and applications.

In the past four decades, numerous studies and efforts have been dedicated to incorporating lignin or lignin derivatives into commercial polymeric materials. The use of multifunctional lignin macromolecules or oligomers as the replacement of polyols is one of the more promising strategies that would enable the use of lignin in a variety of applications in adhesives [130], foams [131] and coatings. However, the immiscibility of lignin and its thermal charring properties has some drawbacks. There are many reports that demonstrate the utility of polymer grafting to improve lignin miscibility and tune thermal properties. Such advances will lead to the utilization of lignins as functional fillers and reinforcement agents to improve the mechanical and thermal properties of polymeric materials. 3D-printable resins made of lignin have gained tremendous interest [132]. Melt stability, extrudability, and mechanical properties are several aspects of investigations that are needed in this research focus. Lignin-based materials such as Arboform™ thermoplastic material by Tecnaro have captured industrial attention. It is essential to have high glass transition temperatures (>60 °C) to replace commodity plastics in the amorphous state [133]. High temperature-resistant thermoplastics, thermosets, and heat-resistant additives are several classes of materials that can be developed from lignin. For example, lignin-derived methacrylate polymers can have tunable and high glass transition temperatures [47,134]. Lignin and its tailor-made derivatives can have many beneficial characteristics for biomedical applications. In several cases, pharmacological activities of lignin such as antiviral, antidiabetic, and antitumor effects are observed [135]. Lignin-based polymeric materials have potential applications in drug delivery and gene delivery as well. Furthermore, lignin is considered to be a useful substrate to produce carbon materials such as activated carbon, carbon fibers, graphitic carbons, and carbon black with applications such as environmental protection, catalysts, energy storage and reinforcing agents [136]. Scion and Revolution Fibers Ltd. (Auckland, New Zealand) have successfully produced lignin-derived continuous carbon nanofiber mats using pilot-scale electrospinning [137]. Lignin-based nanomaterials are gaining interest. Nanolignin-derived materials may provide new approaches for polymeric lignin valorization [138].

Interest should also be given to understanding the environmental impact of lignin extraction via the paper pulping process or lignin-first methods. Since lignin is an important soil former, one aspect of research is to understand how much wood or lignin should be present in natural environments for biodegradation and soil ecosystem balance. Many researchers are interested in exploring microorganisms such as white rot fungi that can efficiently degrade woody material and lignin. There is evidence to support the biodegradation of lignin, and that lignin biodegradation can be accelerated using other carbon sources for microorganisms. However, it would be interesting to study how polymer grafting can be used as a tool to fine tune lignin biodegradation.

The biotechnology advances to produce designer lignins, as well as improvements in lignin isolation methods, will enhance lignin utilization as a chemical feedstock. Increased environmental awareness and education change consumer behavior towards “greener” materials that may help biobased materials compete with petroleum-based products. However, it is always challenging to replace current commodity materials without a performance advantage. Given the competitive nature of petroleum-based alternatives, lignin-based new chemicals and materials need careful and cost-effective designs with reproducible properties in order to reach into the market to serve as value-added materials. Lignin valorization requires combined global efforts from scientists representing many fields of science and engineering. With more efforts from the global research community, more advanced, high-performance lignin biopolymers and composites will be a technical and commercial success in the near future.

## Figures and Tables

**Table 1:** Major extraction methods to obtain lignin or lignosulfonates. Adapted with permission [4,18,20].

Extraction Method	Extraction Conditions	Lignin Properties	Remarks
Kraft Pulping Process	Wood chips are digested in aqueous NaOH and Na_2_S at 150–170 °C for 2 h. This breaks down lignin and solubilizes it. After the cellulose fibers are recovered, lignin is precipitated by lowering the pH of the soap-free black liquor.	Soluble in alkali media and some organic solvents (DMSO, DMF, pyridine) Molecular weight 1000–15,000 g/mol, *Đ* 2.5–3.5, sulfur 1–3%, ash 0.5–3%, *T*_g_ 140–160 °C.	The globally dominant method for isolating lignin from paper pulping waste. It is estimated that more than 20 million tons of kraft lignin are produced in the United States [21]. Mostly sugar-free lignin with some condensed and –SH group attached structures are obtained by this process. All types of wood and non-wood species like bamboo can be used as the substrate for kraft pulping process. Reactive sites are present for sulfonation or other chemistries. However, large volumes of kraft lignin are used as boiler fuel in paper mills. Westvaco (now Ingevity Corporation) developed the initial patented kraft lignin recovery process [22]. More recently, Lignoboost [23] and LingoForce [24] processes are developed that enable integrated lignin isolation.
Sulfite Process	140–170 °C, H_2_O, metal sulfites (e.g., Na_2_SO_3_, NaHSO_3_, (NH_4_)_2_SO_3_, MgSO_3_, CaSO_3_) and sulfur dioxide, 1–5 h	Soluble in water, molecular weight 1000–50,000 g/mol, *Đ* 6–8, sulfur 4–8%, ash 4–8%, *T*_g_ ~ 130 °C.	Lignosulfonates are obtained with highly condensed structures and –SO_3_ groups. An estimated 1.5 million tons of sulfite lignin is annually produced. Higher in sugar content and impurities. Mostly used as a cement additive. Less control is available over the location of sulfonate groups or the degree of sulfonation.
Soda Lignin	120–170 °C, H_2_O, NaOH, anthraquinone as a catalyst	Soluble in alkali media, molecular weight 1000–3000 g/mol, *Đ* 2.5–3.5, sulfur-free, ash 0.7–2.3%, *T*_g_ ~ 140 °C.	Soda lignin is sulfur free and has less condensed structures. An estimated
Organosolv Lignin	Organic solvents such as alcohol or alcohol/water mixtures, formic acid, and acetic acid. Treated at 170–190 °C.	Soluble in alkali media, molecular weight 500–5000 g/mol, *Đ* 1.5–2.5, sulfur-free, ash 1.7%, *T*_g_ ~ 100 °C.	Organosolv lignin is obtained sulfur-free with relatively high purity. This is a mild process that results in less structural modifications.

**Table 2:** Summary of recent literature on ATRP modifications of lignin.

Lignin Source	Monomers (Representative)	Catalyst system and Conditions	Application	Ref.
Kraft lignin from Westvaco Corp (Ingevity Corp) (Charleston, SC)	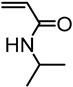	ATRP: CuBr/PMDTA in water/DMF at 50 °C	Thermoresponsive materials	[72]
Organosolv lignin, Lignol Corporation	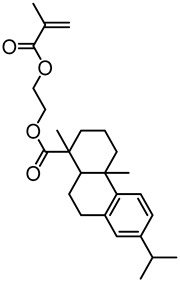	ATRP: CuBr/Me_6_TREN in THF at 65 °C	Hydrophobic polymer composites	[73]
Kraft lignin from Westvaco Corp (Ingevity Corp) (Charleston, SC)	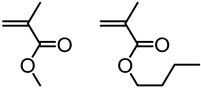	ATRP: CuBr/PMDTA in water/DMF at 80 °C	Thermoplastic elastomers	[74]
Softwood Kraft lignin, Ingevity Corp. (Charleston, SC)	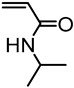	SI-ATRP: CuCl/HMTETA in water at room temperature	Ionic-responsive nanofibrous mats	[75]
Kraft lignin (alkali), Tokyo Chemical Industry, Co.	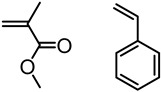	ATRP: CuCl/bpy or CuBr/PMDETA in DMF at 100 °C	Thermoplastic lignin composites	[76,77]
Kraft lignin (alkali), Sigma-Aldrich	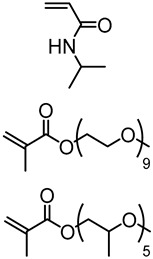	ATRP: CuBr/HMTETA in 1,4-dioxane at 60–70 °C	Thermogelling copolymers	[78]
Kraft lignin (alkali), Shuntai Technology Corp. (Huaihua, Hunan, China)	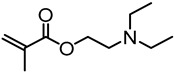	ATRP: CuBr/PMDTA in DMF at 70 °C	CO_2_ responsive nanoparticles for Pickering emulsions	
Kraft lignin (alkali), Sigma-Aldrich	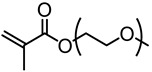	ATRP: CuBr/HMTETA in acetone at room temperature	Supramolecular hydrogels/self-healing materials	[79]
Lignin, Tokyo Kasei Kogyo Co., Ltd.	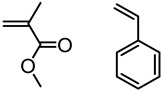	AGET ATRP: FeCl_3_.6H_2_O/PPh_3_/ascorbic acid in DMF at 110 °C for St and 90 °C for MMA	Novel polymerization method	[80]
Kraft lignin (alkali), Sigma-Aldrich	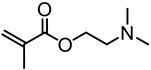	CuBr/HMTETA in 1,4-dioxane at 65 °C	Gene delivery	[81]

**Table 3:** Summary of recent literature on RAFT grafting from modifications on lignin.

Lignin Source	Monomers (Representative)	RAFT CTA and Conditions	Application	Ref.
Kraft lignin (alkali), Tokyo Chemical Industry, Co.	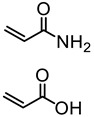	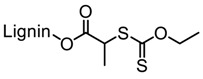 AIBN/DMF at 70 °C	Surfactants, Pickering emulsions, Cement superplasticizers	[85,86,87]
Kraft lignin (alkali), Tokyo Chemical Industry, Co.	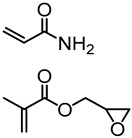	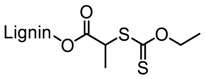 AIBN/DMF at 70 °C	High-performance superplasticizers	[88]
Organosolv lignin, Lignol Corporation	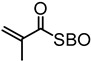	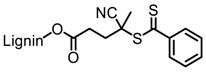 AIBN/toluene at 70 °C	Biocomposites	[89]
Kraft lignin, Guangxi Nanning Phoenix Pulp & Paper Co., Ltd., China	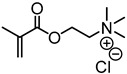	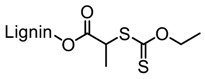 AMBN in DMF/water at 70 °C	Cationic flocculant	[90]

**Table 4:** ROP literature for the preparation of lignin copolymers.

Lignin Source	Monomers (Representative)	Initiator and Conditions	Application	Ref.
Lignophenol from Japanese cedar	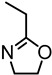	Benzyl bromide moieties, bulk, 100 °C, 12 h	Composites	[91]
Sulfonated lignin from Sigma Aldrich, USA	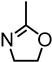	Tosylated lignin, DMSO, 100 °C, 10 h	Anti-infective ointment	[92]
Indulin AT from Ingevity Corp. (Charleston, SC)	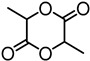	Lignin hydroxyls, triazabicyclodecene (TBD), bulk, 130 °C for 3.5 h	Composites	[93]
Biobutanol lignin from Songyuan bairui bio-polyols Co. Ltd.	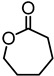	BBL initiator, triazabicyclodecene (TBD), bulk, 135 °C	Composites, coatings	[94]
Alkali lignin or organosolv lignin from Sigma Aldrich,	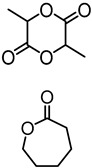	Sn(Oct)2, 120–130 °C, 24–45 h, solvent free or with solvents such as toluene	Composites, nanofibers for healthcare applications	[95,96]
Alkali lignin	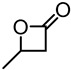	solvent-free ROP, tin(II) 2-ethylhexanoate, 130 °C for 24 h	Nanofibers for biomedical applications	[97]
Softwood Kraft lignin from UPM BioPiva	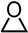	Hydrogenolyzed lignin hydroxyls, Phosphazene base P4-t-Bu, THF, at 50 °C for 2 days	Non-ionic surfactants	[98]

## Abbreviations

ADMET	Acyclic diene metathesis
AGET ATRP	Activators generated by electron transfer atom transfer radical polymerization
AIBN	Azobisisobutyronitrile
ATRP	Atom transfer radical polymerization
BBL	Biobutanol lignin
BiBB	2-Bromoisobutyryl bromide
CTA	Chain transfer agent
CRP	Controlled radical polymerization
DEAEMA	2-(Diethylamino)ethyl methacrylate
DMAEMA	2-(Dimethylamino)ethyl methacrylate
DMPA	2,2-Dimethoxy-2-phenylacetophenone
FRP	Free radical polymerization
HLS	Hardwood lignosulfonates
KF-3CR	Kabachnik-Fields three-component reaction
LCST	Lower critical solution temperature
LMC	Lignin model compound
LP	Lignophenol
MCR	Multi-component reaction
NIPAM	*N*-isopropylacrylamide
NMP	Nitroxide mediated polymerization
PAA	Poly(5-acetylaminopentyl acrylate)
PC	Polycarbonate
PCL	Polycaprolactone
PEG	Poly(ethylene glycol)
PET	Poly(ethylene terephthalate)
PHA	Polyhydroxyalkanoate
PHB	Poly(3-hydroxybutyrate)
PLLA	Poly(l-lactide)
PNIPAAm	Poly(*N*-isopropylacrylamide)
PPG	Poly(propylene glycol)
PSA	Pressure-sensitive adhesives
PSt	Polystyrene
PVC	Poly(vinyl chloride)
PVP	Polyvinylpyrrolidone
RAFT	Reversible addition fragmentation chain transfer
ROMP	Ring-opening metathesis polymerization
ROP	Ring-opening polymerization
SI-ATRP	Surface-initiated ATRP
SLS	Softwood lignosulfonates
TAAC	Thermal azide−alkyne cycloaddition reaction
TBD	Triazabicyclodecene
TGA	Thermal gravimetric analysis
TPE	Thermoplastic elastomers

## Appendix A

**Dr. Mitra S. Ganewatta** received his B.Sc. (honors) in chemistry from University of Colombo, Sri Lanka. He obtained his Ph.D. in chemistry under the advice of Prof. Chuanbing Tang at the Department of Chemistry and Biochemistry, University of South Carolina. His doctoral work included biomass-derived polymers and biomaterials development. He was a postdoctoral fellow at the University of Minnesota, with Professor Theresa M. Reineke. He joined Ingevity Corporation, South Carolina in 2018. Currently, he serves as a lead development chemist in the Industrial Specialties division with a research focus on developing high-value products from pine tree derived chemicals rosin, tall oil fatty acids, and lignin.

**Dr. Hasala N. Lokupitiya** obtained her B.Sc. (honors) in chemistry from University of Colombo, Sri Lanka. She received her Ph.D. in chemistry under the supervision of Prof. Morgan Stefik at the Department of Chemistry and Biochemistry, University of South Carolina. Her Ph.D. research focus was on nanomaterial development using polymeric templates. Then, she worked with Prof. Jane E. Wissinger at the University of Minnesota to develop green chemistry routes for polymer synthesis. Currently, Dr. Lokupitiya works as an Adjunct Professor at the College of Charleston, South Carolina.

**Professor Chuanbing Tang** received his B.S. from Nanjing University and Ph.D. from Carnegie Mellon University with Profs. Krzysztof Matyjaszewski and Tomasz Kowalewski. He was a postdoctoral scholar at the University of California Santa Barbara with Profs. Edward J. Kramer and Craig J. Hawker. Since August 2009, he has been an Assistant, Associate, full professor and College of Arts and Sciences Distinguished Professor in Department of Chemistry and Biochemistry at the University of South Carolina. His research interests focus on organic polymer synthesis, sustainable polymers from renewable natural resources, metal-containing polymers, polymers for biomedical application and nanophase-separated copolymers for energy storage. He has been recognized with several awards including NSF Career Award and Presidential Early Career Awards for Scientists and Engineers (PECASE). He is a Kavli Fellow of National Academy of Sciences and POLY Fellow of American Chemical Society. He has published over 100 papers and 15 patent applications.
